# Bacterial bioactive metabolites can facilitate cooperative interactions within *Aspergillus fumigatus–Pseudomonas aeruginosa* mixed biofilms by promoting fungal polysaccharide production

**DOI:** 10.1128/aem.00918-26

**Published:** 2026-06-15

**Authors:** Qiuchen Li, Huike Zhang, Xiao Shu, Longyun Cong, Wei Wang, Shizhu Zhang

**Affiliations:** 1 Jiangsu Key Laboratory for Pathogens and Ecosystems, Jiangsu Engineering and Technology Research Center for Microbiology, College of Life Sciences, Nanjing Normal University, Nanjing, China; Royal Botanic Gardens Kew, Surrey, United Kingdom

**Keywords:** *Aspergillus fumigatus*, *Pseudomonas aeruginosa*, phenazines, rhamnolipids, galactosaminogalactan

## Abstract

Pathogens are often present alongside other microbial species in chronic infections. The mold *Aspergillus fumigatus* and bacteria *Pseudomonas aeruginosa* are both prevalent opportunistic pathogens in cystic fibrosis (CF) patients, and their interactions play a role in the pathogenesis of chronic airway disease. In this study, their interactions were investigated during the formation of *A. fumigatus–P. aeruginosa* mixed biofilms. *P. aeruginosa* is capable of differentially modulating mixed biofilm formation, shifting from suppressing fungal growth to promoting robust mixed biofilm formation as the concentration of *P. aeruginosa* decreases. Bioactive secondary metabolites derived from *P. aeruginosa*, such as phenazines and rhamnolipids, have dual roles on fungal development and mixed biofilm formation. At high concentrations, these metabolites inhibit fungal growth, whereas at low concentrations, they promote the production of the fungal polysaccharide GAG; this, in turn, modulates mixed biofilm formation and enhances both bacterial biomass and drug resistance in the mixed biofilms. Both phenazines and rhamnolipids can induce the production of mitochondria-derived reactive oxygen species (ROS), which in turn act as signaling molecules to enhance GAG production. Finally, mitogen-activated protein kinase A (MpkA) was identified to mediate the response to bacterial metabolite-induced ROS and the enhancement of GAG production. Bacterial bioactive metabolites facilitate cooperative interactions with fungi via a conserved stress response pathway.

**IMPORTANCE:**

The secondary metabolites of *Pseudomonas aeruginosa*, including phenazines and rhamnolipids, are traditionally regarded as toxins that inhibit the growth of competing microorganisms. Here, it is demonstrated that low doses of phenazines and rhamnolipids trigger the pathogenic fungus *Aspergillus fumigatus* to produce polysaccharide GAG, which in turn promotes robust *A. fumigatus–P. aeruginosa* mixed biofilm formation and subsequently enhances bacterial biomass and drug resistance. Mitogen-activated protein kinase A (MpkA) plays a crucial role in this process. Collectively, these bioactive metabolites play a previously unrecognized role in the interactions of human fungal pathogens. This provides a foundation for understanding interspecies signaling mechanisms in clinical biofilms.

## INTRODUCTION

Biofilms are complex microbial communities that grow while encased within an extracellular matrix. A key hallmark of biofilms is their enhanced resistance to antimicrobial agents and host immune defenses, a trait that renders them notoriously difficult to eradicate (1, 2). *Pseudomonas aeruginosa*, a Gram-negative bacterium, is a common cause of respiratory tract infections in adult patients with chronic pulmonary diseases (3, 4). *Aspergillus fumigatus* is a prevalent airborne, filamentous fungal pathogen that induces a spectrum of diseases, including invasive aspergillosis (IA) and chronic pulmonary aspergillosis (5, 6). These organisms are frequently observed to co-colonize in the lungs of cystic fibrosis (CF) patients (7–9), and such co-colonization has been consistently linked to declines in pulmonary function (7). The frequent co-isolation of these two microbes suggests that fungal–bacterial interactions may contribute to disease pathogenesis.

The interaction between *P. aeruginosa* and *A. fumigatus* has been extensively investigated under vitro conditions. Recently, a subset of secondary metabolites has been identified within *A. fumigatus–P. aeruginosa* mixed biofilms (10, 11). These secondary metabolites are thought to play potential roles in interspecies interaction and mixed biofilm formation. Indeed, the *A. fumigatus*-derived secondary metabolite pyripyropene plays an important role in *A. fumigatus–P. aeruginosa* mixed biofilm formation (11). In contrast, phenazines, as well as different analogs of the siderophore pyoverdin and rhamnolipids, were identified as the primary compounds produced in *A. fumigatus–P. aeruginosa* mixed biofilms (10). Phenazines comprise a major category of bacterial secondary metabolites and are nitrogen-containing aromatic compounds with diverse physiological functions (12–14). *P. aeruginosa* produces several phenazines, including pyocyanin (PYO), phenazine-1-carboxylic acid (PCA), phenazine-1-carboxamide (PCN), 5-methyl-phenazine-1-carboxylic acid (5-Me-PCA), and 1-hydroxyphenazine (1-HP) (10, 13). Phenazines exert significant inhibitory effects on fungal growth, and their toxicity is thought to arise, at least in part, from redox activity and the concomitant production of reactive oxygen species (ROS) (13, 15, 16). Furthermore, the surfactant dirhamnolipids suppress fungal growth through inhibition of β-1,3-glucan synthase, a crucial enzyme for fungal cell wall biosynthesis (17), while the siderophore pyoverdine sequesters iron and impairs fungal growth (18, 19).

Although multiple secondary metabolites of *P. aeruginosa* exert inhibitory effects on fungi, these toxin secondary metabolites can exert alternative effects on fungal development under specific conditions. In the setting of bacterial-yeast biofilms, moderate concentrations of phenazines can regulate the morphological development of *Candida albicans* biofilms through alteration of fungal respiratory activity (20). Furthermore, phenazines can modulate *Aspergillus* development, shifting fungal growth from weak vegetative states toward induced asexual sporulation (conidiation) as the concentration of phenazines decreases (21). In addition, sub-inhibitory concentrations of PYO, PCA, and PCN can promote the growth of *A. fumigatus* by facilitating iron acquisition, potentially through reduction of iron to the more bioavailable ferrous form (16, 22, 23). Therefore, the outcome of fungal-bacterial interactions may depend on their relative ratios during co-colonization and on the local concentrations of these secondary metabolites.

The initial stages of *A. fumigatus-P. aeruginosa* interaction are driven by the adherence of *P. aeruginosa* to the hyphal surface. Fungal glycosaminoglycans (GAG) are critical for this process, as *P. aeruginosa* can attach to *A. fumigatus* hyphae in a GAG-dependent manner (17, 24). GAG is a heteropolysaccharide composed of α-1,4-linked galactose and partially deacetylated N-acetyl galactosamine (GalNAc) (25, 26). It functioned as a major adhesin in *A. fumigatus*, with multiple roles associated with biofilm formation and host tissue invasion (27–29). Therefore, GAG plays a crucial role not only in fungal biofilm formation but also in interactions between *P. aeruginosa* and *A. fumigatus*. Importantly, GAG production is highly plastic, as cell wall stresses imposed by CFW or antifungal agent caspofungin, which inhibits 1,3-β-glucan synthase, can stimulate GAG production (30). However, it remains unclear whether GAG production can be regulated by biological factors—for instance, whether bacteria are capable of modulating GAG production.

In this study, we reveal a dual role of *P. aeruginosa*-derived bioactive metabolites phenazines and rhamnolipids on fungal development and mixed biofilm formation. At high concentrations, these metabolites inhibit fungal growth, whereas at low concentrations, they promote mixed biofilm formation. These findings uncover a novel mode of action of these bioactive metabolites and their impact on microbial interactions.

## RESULTS

### Moderate concentrations of *P. aeruginosa* promote the formation of robust *A. fumigatus–P. aeruginosa* mixed biofilms

**Fig 1:**
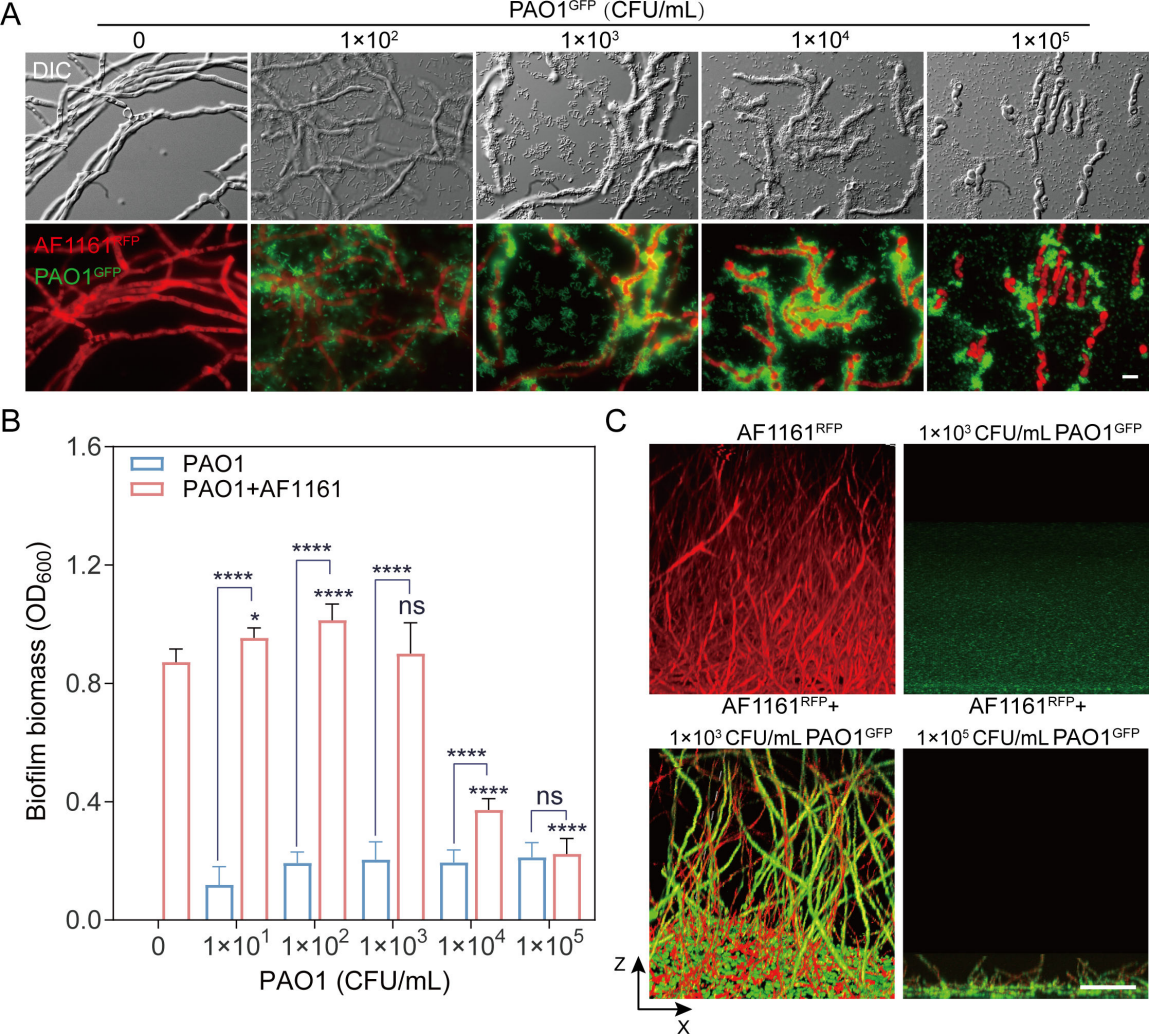
*A. fumigatus* can form robust mixed biofilms with *P*. *aeruginosa* at moderate concentrations. (**A**) Representative images showing the growth status of AF1161^RFP^ co-cultured with PAO1^GFP^ at different concentrations. 1 × 10^5^ conidia/mL AF1161^RFP^ and PAO1^GFP^ at indicated concentrations were statically cultured in RPMI-1640 medium at 37°C for 12 h. Scale bar = 10 µm. (**B**) The biomass assay of AF1161 biofilm alone and the mixed biofilm biomass. 1 × 10^5^ conidia/mL AF1161 with or without PAO1 at indicated concentrations were statically cultured in RPMI-1640 medium at 37°C for 24 h. The biofilm biomass was determined by crystal violet assay. Bars represent mean ± standard deviation (SD). (**C**) Representative laser scanning confocal images of biofilm morphology of AF1161^RFP^ alone and in mixed culture with PAO1^GFP^ at indicated concentrations. 1 × 10^5^ conidia/mL AF1161^RFP^ and PAO1^GFP^ at indicated concentrations were statically cultured in RPMI-1640 medium at 37°C for 24 h. Scale bar = 100 µm. All experiments shown above were performed three times. A two-way analysis of variance (ANOVA) along with Šidák multiple comparison tests was utilized for statistical analysis (**, *P* < 0.01; ***, *P* < 0.001; ****, *P* < 0.0001; ns, no significant difference).

To determine the influence of *P. aeruginosa* on *A. fumigatus* development, a series of concentrations of green-fluorescent protein (GFP)-producing *P. aeruginosa* strain PAO1 (PAO1^GFP^) ranging from 1 × 10^2^ to 1 × 10^5^ CFU/mL were simultaneous static cocultures with 1 × 10^5^ conidia/mL red fluorescent protein (RFP)-producing *A. fumigatus* strain AF1161 (AF1161^RFP^). Microscopic observations indicated that relatively lower concentrations of *P. aeruginosa* (1 × 10^2^ and 1 × 10^3^ CFU/mL) did not significantly affect fungal growth. Conversely, *P. aeruginosa* at higher concentrations (1 × 10^4^ and 1 × 10^5^ CFU/mL) markedly inhibited fungal growth (Fig. 1A). In addition, abundant *P. aeruginosa* cells were observed adhering to *A. fumigatus* hyphae. It has been previously reported that fungal GAG is required for *P. aeruginosa* adherence to *A. fumigatus* hyphae (24). Consistent with these reports, abundant *P. aeruginosa* was not observed on the hyphae of ∆*uge3* mutant (Fig. S1). The *uge3* gene encodes a glucose 4-epimerase required for GAG production (28).

The biomass of *A. fumigatus–P. aeruginosa* mixed biofilms was assayed using crystal violet absorbance. Mixed biofilms produced substantially greater biomass than bacterial monoculture biofilms. In comparison, mixed biofilms produced more or comparable biomass relative to fungal monoculture biofilms when *A. fumigatus* was co-cultured with relatively low concentrations of *P. aeruginosa* (1 × 10^2^ and 1 × 10^3^ CFU/mL) (Fig. 1B). In contrast, increasing concentrations of *P. aeruginosa* (1 × 10^4^ and 1 × 10^5^ CFU/mL) significantly reduced mixed biofilm biomass to levels comparable to those of *P. aeruginosa* monoculture biofilms (Fig. 1B). Considering that changes in crystal violet absorbance cannot be straightforwardly attributed to mixed biofilm promotion or inhibition, we used confocal laser scanning microscopy (CLSM) to observe biofilm formation by *P. aeruginosa* and *A. fumigatus*. CLSM images revealed that *P. aeruginosa* formed a thin and loose biofilm when cultured alone. However, when relatively low concentrations of *P. aeruginosa* (1 × 10^3^ CFU/mL) were co-cultured with *A. fumigatus*, *P. aeruginosa* adhered to fungal hyphae and further developed into a robust mixed biofilm. Under these conditions, *A. fumigatus* growth is not significantly inhibited, and *P. aeruginosa* efficiently adheres to fungal hyphae. In contrast, *P. aeruginosa* at 1 × 10^5^ CFU/mL greatly inhibited *A. fumigatus–P. aeruginosa* mixed biofilm formation (Fig. 1C).

Overall, the above results demonstrate that the initial concentration of *P. aeruginosa* is a crucial determination of *A. fumigatus–P. aeruginosa* mixed biofilm development. A higher initial concentration of *P. aeruginosa* suppresses *A. fumigatus* growth and inhibits mixed biofilm formation, whereas a relatively lower initial concentration exerts minimal impact on fungal growth while promoting the development of robust *A. fumigatus–P. aeruginosa* mixed biofilms.

### *P. aeruginosa* enhances its biomass and drug resistance in mixed-species biofilms in a GAG-dependent manner

**Fig 2:**
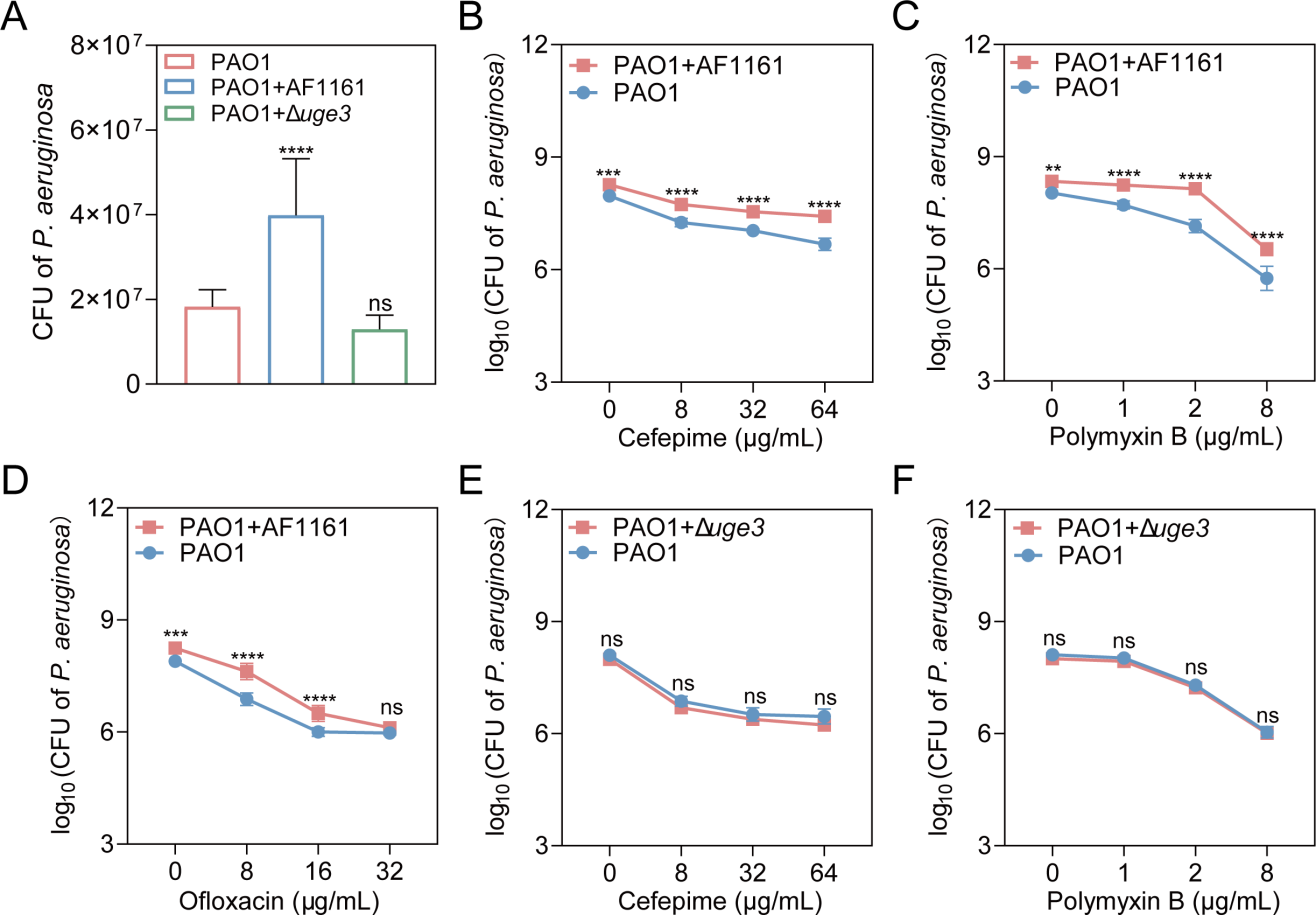
*A. fumigatus* GAG is required for *P. aeruginosa* biomass enhancement and drug resistance in the mixed biofilms. (**A**) CFU counting of *P. aeruginosa* in biofilms. 1 × 10^3^ CFU/mL *P*. *aeruginosa* was statically co-cultured with or without 1 × 10^5^ conidia/mL *A. fumigatus* AF1161 or ∆*uge3* mutant at 37°C for 18 h. The number of *P. aeruginosa* was determined by CFU counting. (**B–F**) Detection of *P. aeruginosa* survival rate in mixed biofilms under indicated antibiotics treatment. 1 × 10^5^ conidia/mL *A. fumigatus* and *P. aeruginosa* at 1 × 10^3^ CFU/mL were statically cultured in RPMI-1640 medium at 37°C for 18 h and then treated with cefepime (**B and E**), polymyxin B (**C and F**), or ofloxacin (**D**) for 6 h. The survival rate of *P. aeruginosa* was determined by comparing the CFU with and without antibiotic treatment. All the above experiments were performed in triplicate. Bars represent mean ± SD. A one-way analysis of variance (ANOVA) along with Dunnett’s multiple comparison tests and two-way ANOVA along with Šidák multiple comparison tests was utilized for statistical analysis (**, *P* < 0.01; ***, *P* < 0.001; ****, *P* < 0.0001; ns, no significant difference).

As described above, *P. aeruginosa* at a moderate concentration (1 × 10³ CFU/mL) forms robust mixed biofilms with *A. fumigatus*. Based on these observations, it was hypothesized that bacterial biomass may be enhanced within mixed biofilms. As expected, the colony-forming unit (CFU) enumeration showed that the number of *P. aeruginosa* cells was increased in mixed biofilms compared with *P. aeruginosa* monoculture biofilms (Fig. 2A), indicating that *P. aeruginosa* benefits from enhanced biomass accumulation in the mixed biofilm. In contrast, this increase in bacterial biomass was not observed when *P. aeruginosa* was co-cultured with the ∆*uge3* mutant (Fig. 2A), indicating that fungal GAG is required for this process.

We further test the killing efficacy of antimicrobial drugs to *P. aeruginosa* in mixed biofilms. A comparison of the effects of several antibiotics on *P. aeruginosa* monoculture biofilms and *P. aeruginosa–A. fumigatus* mixed biofilms, as determined by CFU counting, revealed that *P. aeruginosa* within mixed biofilms exhibited increased resistance to polymyxin B and cefepime, and, to a lesser extent, to ofloxacin, compared with *P. aeruginosa* monoculture biofilms (Fig. 2B through D). Strikingly, the increased drug resistance observed in mixed biofilms was also dependent on GAG, as this effect was abolished when *P. aeruginosa* was co-cultured with the ∆*uge3* mutant (Fig. 2E and F). Collectively, these results suggest that fungus-derived GAG contributes to increased *P. aeruginosa* biomass and protects it from killing by certain antimicrobial agents within *P. aeruginosa–A. fumigatus* mixed biofilms.

### *P. aeruginosa* secondary metabolites phenazines and rhamnolipids are required for the induction of GAG in mixed biofilms

**Fig 3:**
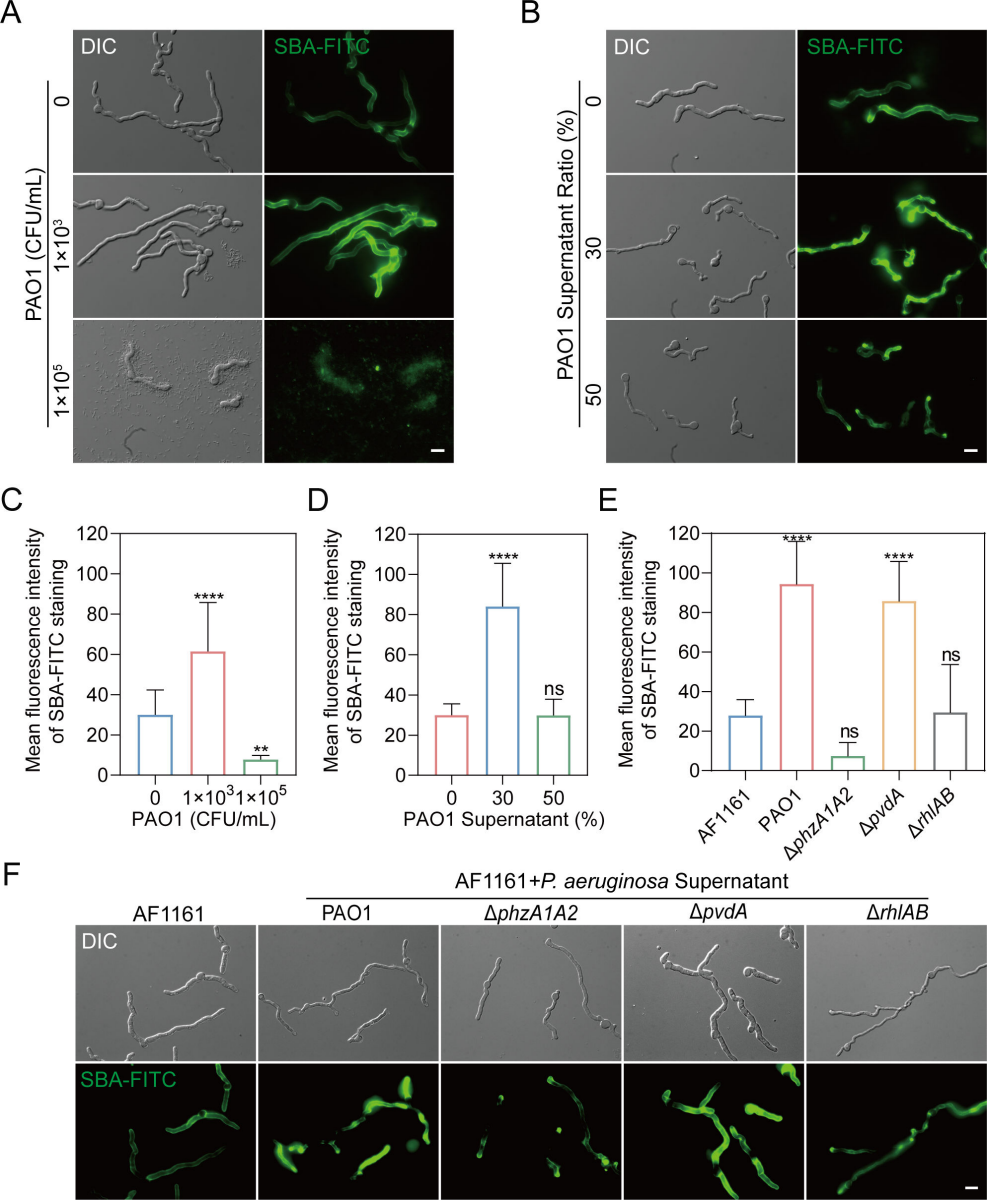
*P. aeruginosa* secondary metabolites phenazine and rhamnolipids are required for GAG induction in *A. fumigatus.* (**A**) Representative images of GAG-specific SBA-FITC staining of *A. fumigatus* co-cultured with *P. aeruginosa*. 1 × 10^5^ conidia/mL *A. fumigatus* and *P. aeruginosa* at indicated concentrations were statically cultured in RPMI-1640 medium at 37°C for 8–10 h and then stained with SBA-FITC. Scale bar = 10 µm. (**B**) Representative images of SBA-FITC staining of *A. fumigatus* co-cultured with *P. aeruginosa* supernatants. 1 × 10^5^ conidia/mL *A. fumigatus* and *P. aeruginosa* supernatant at indicated ratio were statically cultured in RPMI-1640 medium at 37°C for 8–10 h and then stained with SBA-FITC. Scale bar = 10 µm. (**C**) Mean fluorescence intensity of images in panel **A**. (**D**) Mean fluorescence intensity of images in panel **B**. (**E**) Mean fluorescence intensity of images in panel **F**. (**F**) Representative images of SBA-FITC staining of *A. fumigatus* co-cultured with *P. aeruginosa* ∆*phzA1A2*, ∆*pvdA*, and ∆*rhlAB* mutant supernatants. 1 × 10^5^ conidia/mL *A. fumigatus* were statically cultured with *P. aeruginosa* supernatant in RPMI-1640 medium at 37°C for 8–10 h. Scale bar = 10 µm. All the above experiments were performed in triplicate. Bars represent mean ± SD. A one-way analysis of variance (ANOVA) along with Dunnett’s multiple comparison tests was utilized for statistical analysis (****, *P* < 0.0001; ns, no significant difference).

Given the critical role of GAG on the interactions between *A. fumigatus* and *P. aeruginosa*, we hypothesized that *P. aeruginosa* might positively regulate fungal GAG production. To test this hypothesis, GAG-specific fluorescein-tagged soybean agglutinin (SBA-FITC) lectin staining was utilized. The mean fluorescence intensity (MFI) of surface-bound GAG in *A. fumigatus* was significantly increased by 2-fold when the fungus was co-cultured with *P. aeruginosa* at a relatively low concentration (1 × 10^3^ CFU/mL) (Fig. 3A and C). In contrast, co-culture with a higher concentration of *P. aeruginosa* (1 × 10^5^ CFU/mL), which markedly inhibited fungal growth, failed to enhance GAG production and instead suppressed it (Fig. 3A and C)

We further analyzed whether *P. aeruginosa* promotes GAG production through direct contact or via secreted small molecules. To this end, we investigated the effects of *P. aeruginosa* supernatants on GAG production. SBA-FITC staining demonstrated that the supplementation with *P. aeruginosa* supernatants at a 30% ratio significantly induced GAG production. In contrast, a higher proportion of *P. aeruginosa* supernatants (50%) failed to enhance GAG synthesis (Fig. 3B and D). Moreover, *P. aeruginosa* supernatant-induced GAG production depended on the GAG biosynthesis regulators such as SomA and MedA (30), as *P. aeruginosa* supernatants failed to induce those mutant strains to produce GAG (Fig. S2).

Phenazines, pyoverdine, and rhamnolipids were the major compounds produced by *P. aeruginosa* in *A. fumigatus–P. aeruginosa* mixed biofilms (10). To investigate the effects of secondary metabolites on *P. aeruginosa*-induced fungal GAG production, *A. fumigatus* was co-cultured with supernatants of ∆*phz* mutant (missing operons phzA1-G1 and phzA2-G2), which produces no phenazines (31); the ∆*pvdA* mutant, which produces no pyoverdine (32); and the ∆*rhlAB* mutant (missing the promoter of rhlAB), which produces no rhamnolipids (33). The supernatants of the ∆*phz* and ∆*rhlAB* mutants, but not ∆*pvdA* mutant, were unable to induce the overproduction of fungal GAG (Fig. 3E and F). Moreover, supplementation of the supernatants from their respective knockout strains with relatively low concentrations (e.g., 10 μg/mL PCA and 0.75 mg/mL rhamnolipids) partially restored their promoting effects on GAG production, whereas supplementation with relatively high concentrations (e.g., 100 μg/mL PCA and 7.5 mg/mL rhamnolipids) did not exert such a restoring effect (Fig. S3). Collectively, those results indicate that phenazines and rhamnolipids secreted by *P. aeruginosa* are crucial for fungal GAG induction.

### Phenazines and rhamnolipids inhibit fungal growth at high concentrations but increase GAG production at moderate concentrations

**Fig 4:**
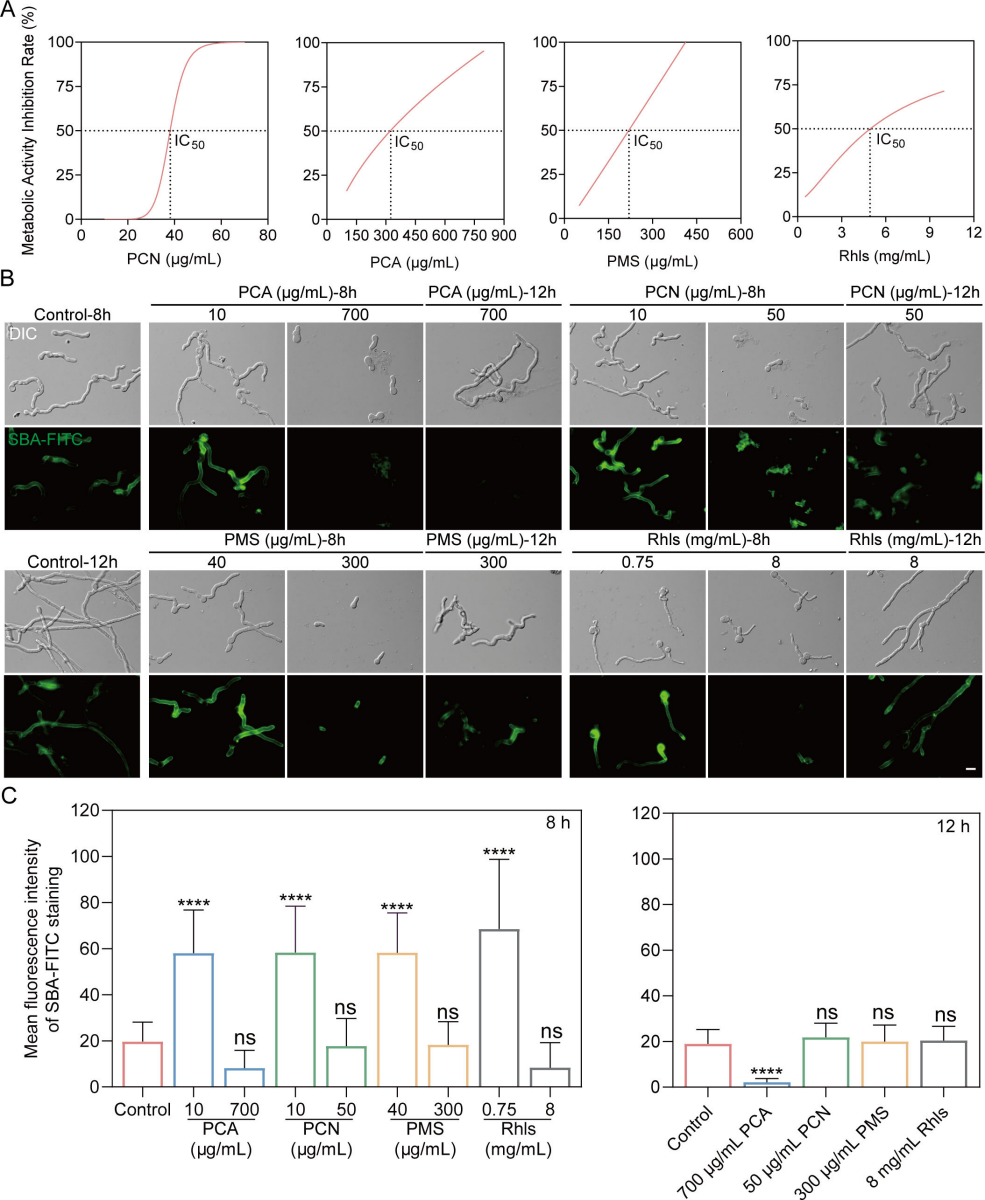
Phenazines and rhamnolipids at high concentrations inhibit fungal growth, but at moderate concentrations increase GAG production. (**A**) Effect of phenazines and rhamnolipids on the metabolic activity of *A. fumigatus*. 1 × 10^5^ conidia/mL *A. fumigatus* were statically cultured with PCN, PCA, PMS, and Rhls in RPMI-1640 medium at 37°C for 12 h. The metabolic activity of *A. fumigatus* after treatment with indicated concentrations of phenazines and rhamnolipids was determined by resazurin assay. (**B**) Representative images of SBA-FITC staining of *A. fumigatus* treated with phenazines and rhamnolipids. 1 × 10^5^ conidia/mL *A. fumigatus* were treated with phenazines and rhamnolipids at indicated concentrations in RPMI-1640 medium at 37°C for indicated time points and stained with SBA-FITC. Scale bar = 10 µm. (**C**) Mean fluorescence intensity of images in panel B. All the above experiments were performed in triplicate. Bars represent mean ± SD. A one-way analysis of variance (ANOVA) along with Dunnett’s multiple comparison tests was utilized for statistical analysis (****, *P* < 0.0001; ns, no significant difference).

To further verify the effects of rhamnolipids and phenazines on fungal development and GAG production, concentration gradients of rhamnolipids and some pure synthetic phenazines, including phenazine-1-carboxylic acid (PCA), phenazine-1-carboxamide (PCN), and phenazine methosulfate (PMS), which has previously been reported as a 5-Me-PCA surrogate (21), on fungal development and GAG production were assayed. We first set out to determine the metabolic inhibitory effects of phenazines and rhamnolipids on *A. fumigatus* using a resazurin-based metabolic activity assay (34). In RPMI-1640 medium (pH = 7.0), the 50% metabolic inhibition concentration (IC_50_) of PCN, PCA, and PMS was determined to be 38 μg/mL, 325 μg/mL, and 225 μg/mL, respectively. Rhamnolipids exhibited the weakest antifungal activity, with an IC_50_ as high as 5 mg/mL (Fig. 4A). Based on these metabolic assays, we selected two concentration ranges of the secondary metabolites: a relatively low concentration causing minimal inhibition of fungal metabolic activity (<10% inhibition) and a relatively high concentration causing substantial inhibition (>50% inhibition). These concentrations were then used to investigate their impacts on fungal development and GAG production. For instance, PCA at 10 μg/mL exerted minimal effects on the hyphal growth of *A. fumigatus*, whereas a higher concentration (700 μg/mL) significantly suppressed hyphal proliferation, as visualized by microscopy (Fig. 4B). Strikingly, SBA staining revealed that low-concentration PCA (10 μg/mL) markedly promoted GAG production in *A. fumigatus*. In contrast, this GAG-promoting activity was abrogated when PCA was used at a high concentration of 700 μg/mL (Fig. 4B and C). In fact, elevated concentrations of PCA exerted an inhibitory effect on GAG biosynthesis. This phenomenon—enhancing GAG production at moderate concentrations while inhibiting fungal growth, with the GAG-inductive effect abolished at high concentrations—was also observed for other phenazines (e.g., PCN, PMS) and rhamnolipids (Fig. 4B and C). This phenomenon was not attributable to reduced hyphal length, as prolonged incubation failed to restore GAG induction by high concentrations of phenazines and rhamnolipids (Fig. 4B and C), indicating that GAG production is not a consequence of altered fungal growth.

Collectively, these findings reveal that phenazines and rhamnolipids secreted by *P. aeruginosa* enhance GAG production in *A. fumigatus* at low concentrations. Conversely, at high concentrations, these metabolites suppress fungal growth and metabolism and simultaneously abrogate their capacity to promote GAG accumulation.

### *A. fumigatus* mitochondrial-derived ROS is essential for the induction of GAG by *P. aeruginosa*

**Fig 5:**
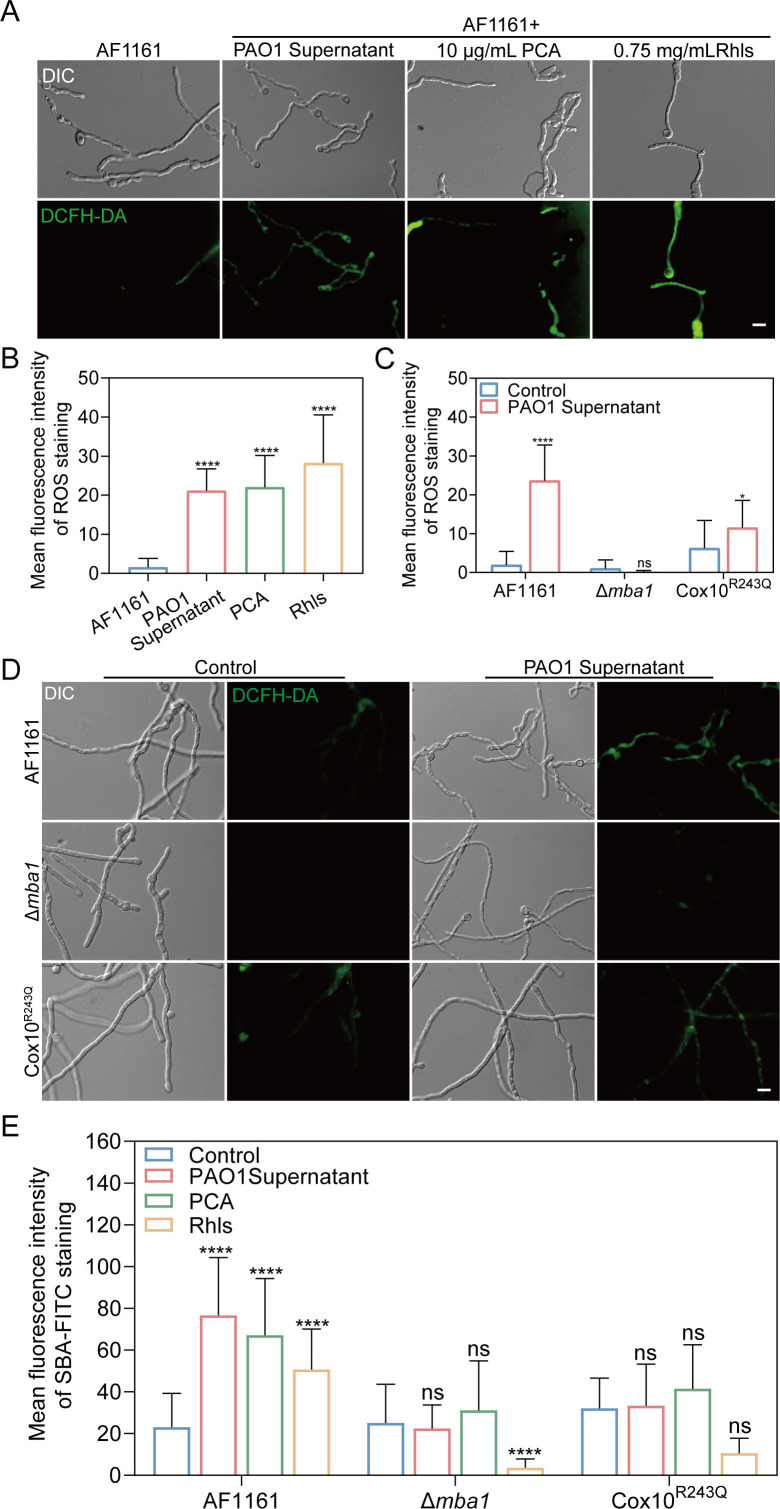
Mitochondrial-derived ROS is required for *P. aeruginosa*-induced GAG production. (**A**) Representative images of DCFH-DA staining of *A. fumigatus* treated with *P. aeruginosa* supernatant, PCA, or Rhls. 1 × 10^5^ conidia/mL *A. fumigatus* were statically cultured with *P. aeruginosa* supernatant, PCA, and rhamnolipids in RPMI-1640 medium at 37°C for 9–11 h. Scale bar = 10 µm. (**B**) Mean fluorescence intensity of images in panel A. (**C**) Mean fluorescence intensity of images in panel D. (**D**) Representative images of DCFH-DA staining of *A. fumigatus* ∆*mba1* and Cox10^R243Q^ mutants treated with *P. aeruginosa* supernatant. 1 × 10^5^ conidia/mL *A. fumigatus* ∆*mba1* and Cox10^R243Q^ mutants were statically cultured with *P. aeruginosa* supernatant in RPMI-1640 medium at 37°C for 9–16 h. Scale bar = 10 µm. (**E**) Mean fluorescence intensity of images of SBA-FITC staining. 1 × 10^5^ conidia/mL *A. fumigatus* ∆*mba1* and Cox10^R243Q^ mutants were statically cultured with *P. aeruginosa* supernatant, PCA, or Rhls in RPMI-1640 medium at 37°C for 8–15 h. Scale bar = 10 µm. All the above experiments were performed in triplicate. Bars represent mean ± SD. A one-way analysis of variance (ANOVA) along with Dunnett’s multiple comparison tests and two-way ANOVA along with Šidák multiple comparison tests was utilized for statistical analysis (*, *P* < 0.05; ****, *P* < 0.0001; ns, no significant difference).

Phenazines can target mitochondria and induce reactive oxygen species (ROS) production in *A. fumigatus* (16). Given that PCA is one of the most abundant phenazines in *A. fumigatus–P. aeruginosa* mixed biofilms (10), we selected PCA as a representative phenazine for subsequent experiments. Consistent with previous reports, either the addition of supernatants of *P. aeruginosa* or the pure synthetic PCA can induce ROS production, as assayed using the fluorescence dye H2DCFDA (Fig. 5A and B). Previous studies have shown that rhamnolipids inhibit fungal growth by suppressing the activity of β-1,3-glucan synthase, which induces cell wall stress (17). Strikingly, rhamnolipids were also found to induce ROS production (Fig. 5A and B).

Despite ROS being toxic at elevated concentrations, moderate ROS levels function as important regulatory signals (35, 36). To test whether the increased ROS is required for *P. aeruginosa*-induced GAG production, the effect of N-acetyl-L-cysteine (NAC), a ROS scavenger, was assessed. NAC completely abolished GAG production induced by supernatants of *P. aeruginosa* or the pure synthetic PCA (Fig. S4A and B). Furthermore, NAC also abolished rhamnolipids-induced GAG production. In addition, relatively low concentrations of the oxidative stressor H_2_O_2_ (1–1.5 μM) can significantly stimulate GAG production in *A. fumigatus* (Fig. S4C and D). These results suggest that oxidative stress generated by rhamnolipids or phenazines acts as a specific signaling cue to promote GAG production.

Mitochondria are the main source of ROS, and because phenazines can target mitochondria, we reasoned that specific mitochondrial components are critical for ROS induction and the subsequent GAG production. Cox10 is a subunit of cytochrome c oxidase (COX), the terminal enzyme of the mitochondrial electron transport chain (complex IV) (37, 38). Mba1 is a mitochondrial membrane-associated protein, and its deletion reduces the activity of complexes I and III of the respiratory chain (39). As expected, ROS production was greatly reduced in the Cox10^R243Q^ mutant and the *mba1* deletion mutant compared with the WT control following treatment with supernatants of *P. aeruginosa* (Fig. 5C and D), indicating that *P. aeruginosa*-induced ROS production depends on mitochondria. A similar phenomenon was observed following rhamnolipid treatment, indicating that rhamnolipid-induced ROS production also occurs in a mitochondria-dependent manner (Fig. S4E and F). Importantly, the increased GAG production was completely abolished in the Cox10^R243Q^ mutant and the *mba1* deletion mutant when co-cultured with *P. aeruginosa* supernatants, rhamnolipids, or the pure synthetic PCA (Fig. 5E; Fig. S4G). Overall, these findings suggest that mitochondrial-derived ROS act as an oxidative stress signal for *P. aeruginosa*-induced GAG production in mixed biofilms.

### *P. aeruginosa*-induced GAG production in *A. fumigatus* is independent of the conserved oxidative stress-responsive transcription factor AfYap1

**Fig 6:**
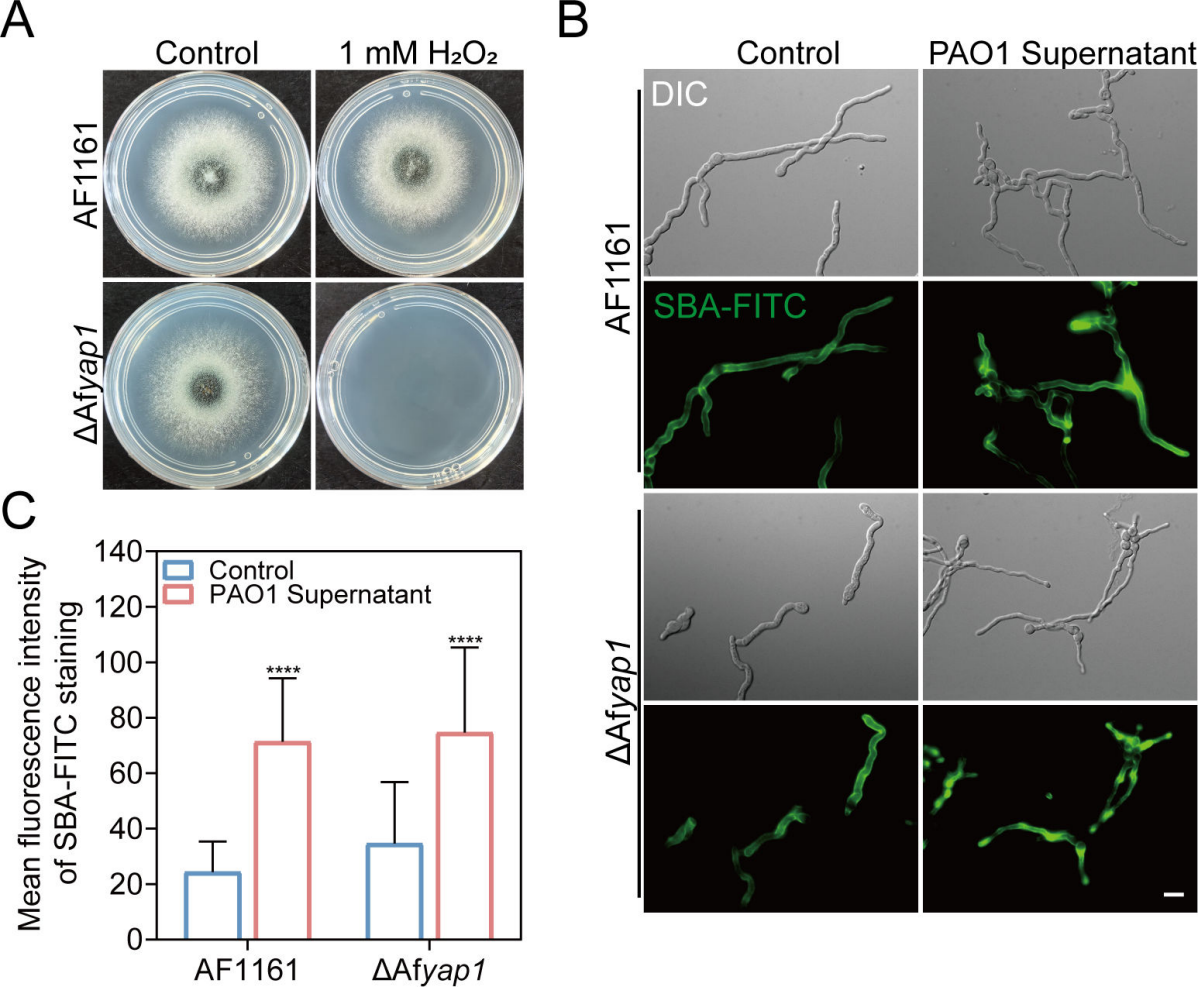
*P. aeruginosa* promotes the increase of GAG in *A. fumigatus* is not associated with *A. fumigatus* Af*yap1*. (**A**) Equal numbers of conidia (2 × 10^4^) of the indicated strains were grown on MM with or without 1 mM H_2_O_2_ at 37°C for 48 h. (**B**) Representative images of SBA-FITC staining of *A. fumigatus* ∆Af*yap1* mutant treated with *P. aeruginosa* supernatant. 1 × 10^5^ conidia/mL *A. fumigatus* were statically cultured with *P. aeruginosa* supernatant in RPMI-1640 medium at 37°C for 8–10 h. Scale bar = 10 µm. (**C**) Mean fluorescence intensity of images in panel **B**. The above experiments were performed in triplicate. Bars represent mean ± SD. A two-way analysis of variance (ANOVA) along with Šidák multiple comparison tests was utilized for statistical analysis (****, *P* < 0.0001).

Having shown that ROS act as an oxidative stress signal for *P. aeruginosa*-induced GAG production in mixed biofilms, we assumed that a fungal oxidative stress response pathway is required for this induction. The bZIP type transcription factor NapA homologs were crucial for oxidative responses from yeast to humans (40, 41). Previously, it has been established that NapA mediates phenazine-triggered oxidative stress signaling to promote asexual development in *A. nidulans* (21). We thus first tested whether AfYap1 (AFUB_075990), a NapA homolog in *A. fumigatus*, participates in *P. aeruginosa*-induced GAG production. Consistent with previous reports (42), the loss of Af*yap1* caused sensitivity to oxidative stressor hydrogen peroxide (Fig. 6A), indicating that AfYap1 is involved in oxidative stress responses in *A. fumigatus*. However, the *P. aeruginosa* supernatants induced GAG production in the ∆Af*yap1* mutant, reaching a comparable level to that of the WT (Fig. 6B and C), indicating that AfYap1 is not involved in *P. aeruginosa*-induced GAG production.

### Mitogen-activated protein kinase A pathway is required for the induction of GAG by *P. aeruginosa*

**Fig 7:**
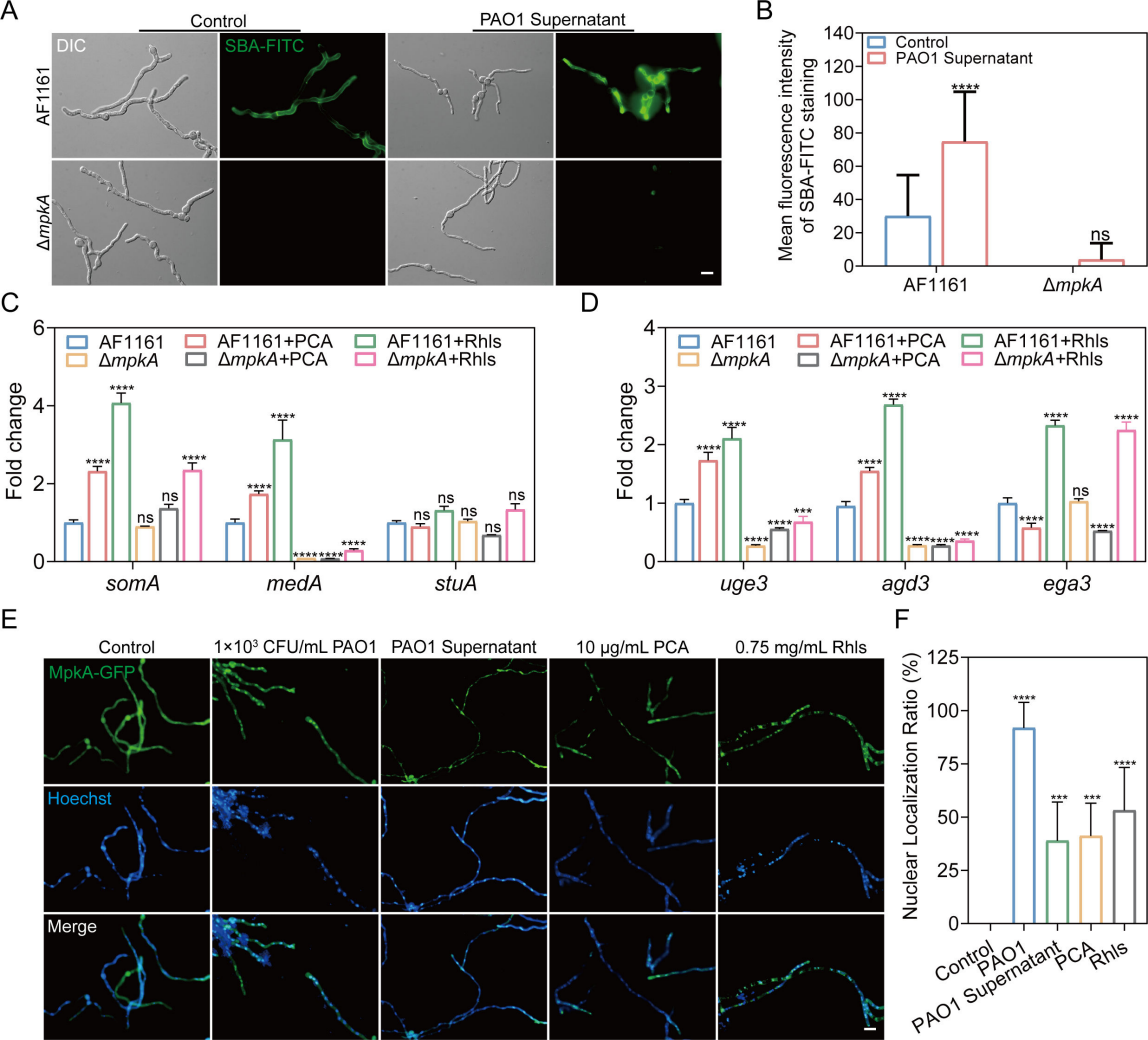
*P. aeruginosa*-induced GAG production is dependent on mitogen-activated protein kinase A. (**A) **Representative images of SBA-FITC staining of *A. fumigatus* ∆*mpkA* mutants treated with *P. aeruginosa* supernatant. 1 × 10^5^ conidia/mL *A. fumigatus* were cultured with *P. aeruginosa* supernatant in RPMI-1640 medium at 37°C for 8–10 h. Scale bar = 10 µm. (**B**) Mean fluorescence intensity of images in panel **A**. (**C and D**). Analysis of the transcriptional expression levels of genes within the GAG biosynthesis gene cluster (**C**) and GAG-related transcription factor-encoding genes (**D**) in *A. fumigatus* under PCA and Rhls treatment. The *A. fumigatus* strains were first cultured statically at RPMI-1640 medium for 12 h, then replaced with fresh medium containing 10 μg/mL PCA or 0.75 mg/mL Rhls, and incubated for another 2 h. Gene expression was normalized to the endogenous reference gene *tubA*, and expression was reported relative to the untreated condition at 12-hour growth. (**E**) Representative images of MpkA-GFP localization. 1 × 10^5^ conidia/mL *A. fumigatus* were incubated with *P. aeruginosa, P. aeruginosa* supernatant, PCA, and Rhls in RPMI-1640 medium at 37°C for 9–11 h, respectively. Nuclear localization was determined by Hoechst staining. Scale bar = 10 µm. (**F**) Quantification of the nuclear localization ratio of MpkA under different treatment conditions. The nuclear import ratio was determined by counting the number of GFP-positive nuclei relative to the total number of Hoechst-labeled nuclei. A total of 15 microscopic images were analyzed for statistical quantification. All the above experiments were performed in triplicate. Bars represent mean ± SD. A two-way analysis of variance (ANOVA) along with Šidák multiple comparison tests was utilized for statistical analysis (***, *P* < 0.001; ****, *P* < 0.0001; ns, no significant difference).

Mitogen-activated protein kinase A (MpkA) is a conserved and essential component of the fungal cell wall integrity pathway (43, 44). Importantly, MpkA is also involved in response to oxidative stress and biofilm formation in *A. fumigatus* (44, 45). Given our observation that mitochondrial ROS production triggered by *P. aeruginosa* secondary metabolites is crucial for GAG biosynthesis, MpkA was reasonably hypothesized to link oxidative stress and GAG production. To test this hypothesis, the ∆*mpkA* mutant was co-cultured with supernatants of *P. aeruginosa*. SBA-FITC staining showed that supernatants of *P. aeruginosa* failed to induce the GAG production in the ∆*mpkA* mutant (Fig. 7A and B). A five-gene cluster has been predicted to encode the enzymes necessary for GAG biosynthesis (46). Real-time PCR showed that rhamnolipids greatly induced the expression of genes within the GAG biosynthesis cluster (including *uge3* and *agd3*) and GAG regulators (including *somA* and *medA*) (fold change > 2, *P* < 0.05). Pure synthetic PCA elicited a similar, albeit less pronounced effect. However, those induced expression of GAG-associated genes was greatly abolished in the ∆*mpkA* mutant (Fig. 7C and D). Collectively, these results demonstrate that MpkA plays a central role in *P. aeruginosa*-induced GAG production in *A. fumigatus*.

The location of MpkA is essential for its activity. Given that MpkA is essential for *P. aeruginosa*-induced GAG production, we assumed that *P. aeruginosa* may affect MpkA translocation. To gain insight into the localization of *A. fumigatus* MpkA, we generated a C-terminally GFP-labeled MpkA (MpkA-GFP) fusion under the control of its own promoter. The growth of the MpkA-GFP strain was comparable to that of the wild type, confirming that the fusion protein is functional (Fig. S5). When grown alone, the fluorescence of *A. fumigatus* MpkA-GFP was evenly distributed throughout the hyphae. In contrast, when co-cultured with *P. aeruginosa* (1 × 10^3^ CFU/mL), the majority of the fluorescence became concentrated within the nuclei (70%) (Fig. 7E and F). The addition of *P. aeruginosa* supernatants (30%), rhamnolipids (0.75 mg/mL), or pure synthetic PCA (10 μg/mL) can also induce the nuclear accumulation of MpkA-GFP (approximately 30%–50%), although the proportion was lower than that in the co-culture with *P. aeruginosa* (Fig. 7E and F). Importantly, the addition of NAC into *P. aeruginosa*, PCA, and rhamnolipids greatly abolished MpkA-GFP accumulation in the nucleus (Fig. S6), suggesting that oxidative stress caused by *P. aeruginosa* is crucial for MpkA-GFP nuclear accumulation. Collectively, the above results demonstrate that MpkA responds to oxidative stress induced by *P. aeruginosa* secondary metabolites, which in turn promotes GAG production in *A. fumigatus*.

## DISCUSSION

**Fig 8:**
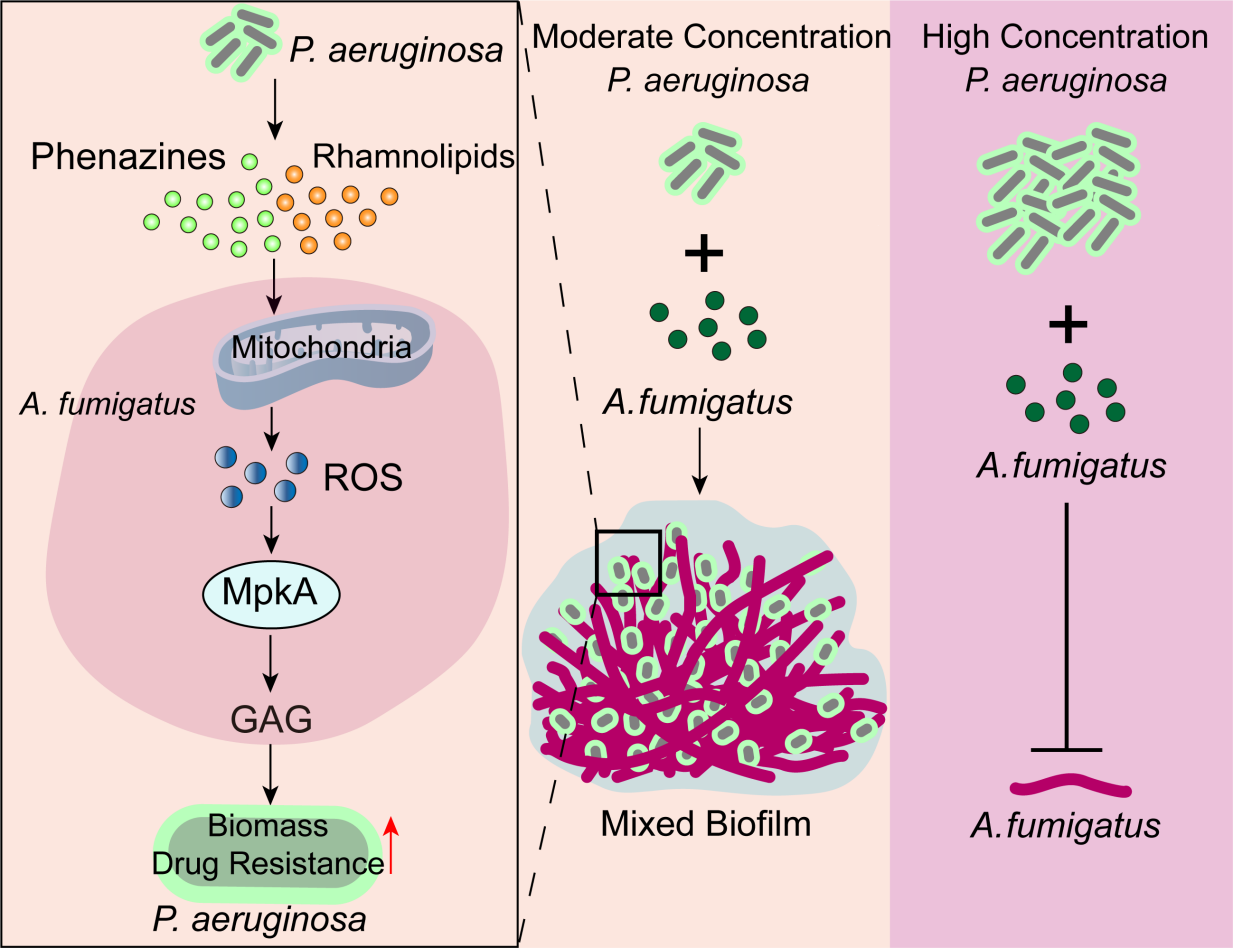
Working model showing the dual roles of *P. aeruginosa* on pathogenic fungi *A. fumigatus* development and mixed biofilms formation. At high concentrations, *P. aeruginosa* inhibits fungal growth; at low concentrations, however, its secreted toxic metabolites, such as phenazines and rhamnolipids, can trigger *A. fumigatus* to produce polysaccharide GAG, which in turn promotes robust *A. fumigatus–P. aeruginosa* mixed biofilm formation and enhances bacterial biomass as well as drug resistance. Mechanistically, the mitogen-activated protein kinase A (MpkA) can respond to ROS induced by phenazines and rhamnolipids, thereby promoting GAG production.

While *P. aeruginosa* secondary metabolites, including rhamnolipids and phenazines, have been reported in previous studies, particularly for their inhibitory effects on fungal growth; their ability to induce GAG biosynthesis and the associated signaling cascade has not been fully addressed. Here, we demonstrate that *P. aeruginosa* metabolites phenazines and rhamnolipids exert concentration-dependent dual effects on *A. fumigatus*: at high concentrations, they inhibit fungal growth, while at low concentrations, they promote production of the fungal exopolysaccharide GAG. This GAG induction, mediated through mitochondria-derived ROS and the MAP kinase MpkA, in turn facilitates robust mixed biofilm formation and enhances bacterial biomass and antibiotic resistance (Fig. 8).

*A. fumigatus* and *P. aeruginosa* exhibit intimate interactions and are ubiquitous components of the pulmonary microenvironment in patients with cystic fibrosis (CF). Most studies investigating interactions between these organisms have demonstrated strong inhibitory effects of *P. aeruginosa* on *A. fumigatus*, particularly on conidial germination (16, 47). However, these findings contrast with clinical observations in which co-colonization is frequently detected in patients with chronic lung diseases. In this study, we identify the initial concentration of *P. aeruginosa* as a key determinant governing these interactions. As the concentration of *P. aeruginosa* decreases, its function shifts from inhibiting fungal growth and mixed biofilm formation to promoting the development of robust mixed biofilms. This finding explains that the complex—either cooperative or inhibitory—interactions between *P. aeruginosa* and *A. fumigatus* observed in varied research systems may arise from differences in their relative abundances in co-culture.

Secreted compounds from *A. fumigatus* and *P. aeruginosa* may exert pleiotropic reciprocal effects on one another in mixed biofilms. Phenazines are the primary secondary metabolites produced by *P. aeruginosa*, and their secretion is elevated when the fungus is present, indicating an important role in interspecies interactions (10). Phenazines have traditionally been recognized as microbial toxins that suppress the growth of competing organisms, including *A. fumigatus* (14, 15), with toxicity thought to arise, at least in part, from redox activity and the associated production of reactive oxygen species (ROS). Indeed, phenazines have redox activity on *A. fumigatus* swollen conidia and hyphae (16). Our results further confirm that phenazines induce mitochondria-dependent ROS production, requiring both Mba1 and Cox10. Despite being toxic at high levels, growing evidence suggests that ROS can serve regulatory functions at moderate levels (35, 36). ROS-mediated signaling has been implicated in multicellular development in bacteria, yeasts, and filamentous fungi. Moderate levels of phenazine-induced ROS have been reported to function as sporulation signals, influencing the development of filamentous fungi within mixed-species communities (21, 35, 48). In this study, we found that moderate concentrations of phenazines and rhamnolipids are capable of inducing the production of fungal polysaccharide GAG. Although rhamnolipids have been shown to target cell wall synthases and potentially induce cell wall stress (17), the abrogation of rhamnolipid-induced GAG production by N-acetylcysteine (NAC) indicates a critical role for oxidative stress in this process. Our findings thus further support a dual role for ROS: acting as cytotoxic agents at high levels while functioning as signaling molecules that promote fungal polysaccharide production at moderate levels.

Notably, GAG induction constitutes a specific response rather than a generic stress reaction. GAG production was abolished by ROS scavenging, mitochondrial dysfunction, or *mpkA* deletion, supporting the involvement of a dedicated signaling cascade rather than a secondary effect of growth inhibition or cell wall damage. Although phenazines and rhamnolipids act through distinct upstream triggers, both signals converge on a common downstream pathway: phenazines target mitochondria to generate ROS, whereas rhamnolipids elicit cell wall stress that signals through mitochondrial ROS. Both inputs ultimately activate MpkA to promote GAG synthesis, explaining their coordinated effect and positioning GAG as a key output of bacterial-fungal cross-talk.

An important finding is that AfYap1, the canonical oxidative stress regulator and NapA homolog in *A. fumigatus*, is dispensable for *P. aeruginosa-*induced GAG production, even though it is required for defense against exogenous H₂O₂. In *A. nidulans*, NapA is essential for phenazine-derived oxidative stress signaling that controls asexual development (21). By contrast, our data reveal that in *A. fumigatus*, MpkA signaling dominates the response to bacterial metabolite-induced ROS, leading to GAG biosynthesis rather than sporulation. This striking divergence highlights *A. fumigatus* perceives and transduces oxidative stress through pathway-specific mechanisms that depend on the downstream biological output.

Moreover, the conserved GAG biosynthetic regulators SomA and MedA were also involved in this process. In our previous study, we found that SomA directly binds to and regulates genes associated with both GAG and cell wall polysaccharide synthesis (30). Consequently, the downstream target genes regulated by both MpkA and SomA include those involved in GAG and cell wall polysaccharide synthesis. However, the relationship between them, such as whether MpkA can directly regulate SomA and MedA, remains to be elucidated.

*P. aeruginosa*-produced phenazines have been detected in the sputum of patients with cystic fibrosis (CF) at concentrations ranging from 1 to 100 μM (approximately 0.2–20 µg/mL for PCA) (49). This range fully overlaps with the low/signaling concentration window employed in the present work, strongly supporting the physiological relevance of the observed inter-kingdom signaling effects. However, the measurements of sputum or tissue homogenates may not reflect metabolite levels within localized infection microenvironments, such as biofilms, bacterial-fungal aggregates, or confined microhabitats (50, 51). As demonstrated in previous co-infection models, *P. aeruginosa* enables to significantly alleviate pulmonary fungal CFU burden, indicating that close pathogen colocalization may facilitate local metabolite enrichment (52). Although the high inhibitory concentrations adopted in experiments (e.g., 700 μg/mL for PCA) are far higher than the typical levels detected in clinical specimens, they are still valuable for delineating the maximum regulatory potential of phenazines against *A. fumigatus* growth. Further studies utilizing animal models of chronic airway co-infection will be required to elucidate the *in vivo* relevance of these *in vitro* findings.

## MATERIALS AND METHODS

### Strains and growth culture conditions

All *A. fumigatus* strains used in this study are summarized in Table S1. *A. fumigatus* strains were cultured on solid YAG medium (glucose, 20 g L^−1^; yeast extract, 5 g L^−1^; 1 mL L^−1^ trace elements, 2% agar) to produce conidia. To establish mixed biofilms, *A. fumigatus* and *P. aeruginosa* co-culture experiments were conducted in RPMI-1640 medium (3-(N-morpholino)propanesulfonic acid [MOPS], 34.53 g L^−1^, RPMI-1640 medium [Sigma-Aldrich, USA], 10.4 g L^−1^, pH 7.0) (53).

### Construction of genetic mutant strains

As previously described, fusion PCR was employed to construct an *mpkA* knockout cassette to generate the *mpkA* deletion strain (54). Briefly, about 1 kb fragments corresponding to the upstream and downstream flanking regions of *mpkA* were amplified using primer pairs ∆*mpkA*-P1/P3 and ∆*mpkA*-P4/P6, respectively. In parallel, the hygromycin B resistance gene (*hph*), used as a selectable marker, was amplified from plasmid pAN7-1 using primers *hph*-F/R. These three PCR products were subsequently used as templates in fusion PCR to generate the *mpkA* knockout fragment using primers ∆*mpkA*-P2/P5. The resulting fusion fragment was then transformed into the parental *A. fumigatus* strain AF1161. Transformants were identified via diagnostic PCR using primer pairs ∆*mpkA*-SF/SR, ∆*mpkA*-P1/*hph*-down, and *hph*-up/∆*mpkA*-P6.

To generate the reporter strain MpkA-GFP, a C-terminal fusion of GFP to MpkA was constructed. Briefly, the *gfp + pyrG* fragment was amplified from plasmid pFNO3 using primers GFP + *pyrG* F/R. Approximately 1 kb fragments flanking the *mpkA* stop codon were amplified using the primer pairs MPKA-P1/P3 and MPKA-P4/P6. These fragments were fused via PCR using primers MPKA-P2/P5, and the resulting fusion product was transformed into strain AF1160. Homologous integration was verified by diagnostic PCR using primer pairs MPKA-P2/GFP + *pyrG* down and GFP + *pyrG*-up/MPKA-P6.

All primers used in this study are listed in Table S2.

### Cultivation of bacterial cultures, colony-forming unit (CFU) counting, and collection of bacterial supernatants

*P. aeruginosa* cultures were grown in RPMI-1640 medium. Single colonies were inoculated into 30 mL RPMI-1640 medium and incubated at 37°C and 220 rpm for 20 h to obtain bacterial cultures. *P. aeruginosa* cultures were serially diluted and plated for colony-forming unit (CFU) enumeration. For *P. aeruginosa* supernatant collection, cultures were centrifuged at 8,000 rpm for 6 min, and the resulting supernatants were filtered through a 0.22 μm membrane filter.

### Biofilm biomass assay

*A. fumigatus* biofilm visualization and quantification were conducted as previously reported (55) with slight modifications. Briefly, *P. aeruginosa* was serially diluted to final concentrations ranging from 1 × 10^1^ to 1 × 10^5^ CFU/mL in RPMI-1640 medium. For mixed biofilm formation, 1 × 10⁵ conidia/mL of *A. fumigatus* were added to each diluted bacterial suspension, followed by static incubation in 24-well plates at 37°C for 24 h. Monoculture *P. aeruginosa* biofilms and *A. fumigatus–P. aeruginosa* mixed biofilms were rinsed twice with 200 μL distilled water. Subsequently, each well was supplemented with 100 μL of 0.1% (wt/vol) crystal violet solution and incubated for 10 min. After staining, 125 μL of absolute ethanol was added to each well for 10 min for decolorization. Finally, 75 μL of the decolorized solution was transferred to a 96-well plate, and the optical density (OD) was measured at 600 nm.

### Laser scanning confocal microscopy (LSCM) of mixed biofilms

Biofilms were cultured for imaging in glass-bottomed cell culture dishes (Φ20 mm, NEST, Wuxi, China). *P. aeruginosa* was cultured at the indicated concentrations, and *A. fumigatus* was cultured at 1 × 10⁵ conidia/mL, either as monocultures or co-cultures, in RPMI-1640 medium at 37°C for 24 h. AF1161^RFP^ and PAO1^GFP^ biofilms were imaged at excitation wavelengths of 532 nm and 488 nm, respectively. Images were captured using a Nikon CFI Plan Apochromat D 10× NA 0.45 objective lens. Time-lapse imaging of the mixed biofilms was performed with z-stack intervals set at 2 μm. All images were acquired and analyzed using NIS Viewer 5.22.

### Determination of the effects of antibiotics against *P. aeruginosa* in biofilms

To assess bacterial survival under antibiotic treatment, *P. aeruginosa* was serially diluted to a final concentration of 1 × 10^3^ CFU/mL in RPMI-1640 medium. For mixed biofilm formation, 1 × 10⁵ conidia/mL of *A. fumigatus* were added to each diluted bacterial suspension. When co-cultured with the *A. fumigatus* ∆*uge3* mutant, the cell culture plates were pretreated with collagen overnight. *P. aeruginosa* monoculture biofilms and *A. fumigatus–P. aeruginosa* mixed biofilms were rinsed with PBS. Subsequently, 500 μL of RPMI-1640 medium containing the different gradients of polymyxin B (A610318, Sangon Biotech), cefepime (C861441, Macklin), or ofloxacin (A600673, Sangon Biotech) was added, while the control group contained drug solvent. The plates were cultured at 37°C for an additional 6 h. After rinsing with PBS, the *P. aeruginosa* monoculture biofilms and mixed biofilms were scraped into a centrifuge tube and resuspended in 1 mL of PBS. The resuspended solutions were serially diluted with RPMI-1640 medium, plated on LB agar plates, and incubated overnight. Bacterial survival rates were determined by comparing the CFU with and without antibiotic treatment, allowing for the assessment of *P. aeruginosa* survival in monoculture and co-culture with *A. fumigatus*.

### Metabolic inhibition rate assay

To evaluate the effects of secondary metabolites on *A. fumigatus* metabolic activity, a resazurin-based assay was conducted as previously reported with slight modifications (34). Resazurin (A606726, Sangon Biotech) was added to a final concentration of 20 μg/mL together with 1 × 10⁵ conidia/mL of *A. fumigatus* in RPMI-1640 medium. Different gradients of PCA (A411018, Sangon Biotech), PCN (A421652, Sangon Biotech), PMS (A610361, Sangon Biotech), or Rhls (R916936, Macklin) were added as indicated. The mixtures were cultured in 96-well plates (200 μL per well) at 37°C for 12 h. Fluorescence intensity (FI) was then measured using an excitation wavelength of 570 nm and an emission wavelength of 590 nm. The metabolic inhibition rate (%) was calculated using the following formula: Metabolic inhibition rate (%) = [1 – (FI_t_ - FI_0_) / (FI_c-_FI_0_)] × 100%, where *FI*_*t*_ refers to the fluorescence intensity of the treated group with phenazines or rhamnolipids; *FI*_*c*_ refers to the fluorescence intensity of the control group without phenazines and rhamnolipids; and *FI*_*0*_ refers to the background fluorescence intensity of resazurin in the presence of the corresponding concentration of phenazines or rhamnolipids.

### Galactosaminogalactan characterization

To characterize galactosaminogalactan (GAG) on the hyphal surface of *A. fumigatus*, an immunofluorescence assay was conducted as previously reported (28, 46). Briefly, *A. fumigatus* was either co-cultured with *P. aeruginosa* or cultured in RPMI-1640 medium supplemented with the indicated reagents. After incubation to obtain hyphae of comparable lengths, hyphal samples were rinsed twice with 500 μL of D-PBS to remove residual substances. The hyphae were then stained with 10 μg/mL SBA-FITC (Vector Labs, Burlingame, CA, USA) under light-protected conditions. This lectin specifically binds to GAG and serves as an indicator of surface-associated GAG abundance. Images were acquired using a Zeiss Axio Imager A1 microscope and arranged with Adobe Photoshop. Individual hyphae were carefully selected in ImageJ (version 1.54p) for quantitative analysis. The final fluorescence value was normalized by calculating the fluorescence intensity divided by the corresponding hyphal area for each selected hypha.

### ROS staining and quantitative analysis

Intracellular reactive oxygen species (ROS) levels were detected using the fluorescent probe DCFH-DA as previously described (16). Briefly, *A. fumigatus* was either co-cultured with *P. aeruginosa* or cultured in RPMI-1640 medium supplemented with the indicated reagents. After incubation to obtain hyphae of comparable lengths, the hyphal samples were rinsed twice with PBS. The hyphae were then stained with 10 μM DCFH-DA (S0033S, Beyotime) at 37°C for 30 min in the dark to allow sufficient probe loading. Fluorescence images were acquired using a Zeiss Axio Imager A1 microscope and processed with Adobe Photoshop. Mean fluorescence intensity was quantified using ImageJ software (version 1.54p)

### MpkA-GFP localization observation

To visualize the subcellular localization of MpkA, the MpkA-GFP strain was either cultured with *P. aeruginosa* supernatants or grown in RPMI-1640 medium supplemented with the indicated reagents. After incubation to obtain hyphae of comparable lengths, samples were rinsed twice with PBS and fixed with 4% paraformaldehyde at room temperature for 45 min. To visualize nuclei, the samples were stained with Hoechst dye (E607328, Sangon Biotech) at a final concentration of 1 μg/mL. Fluorescence images were acquired using a Zeiss Axio Imager A1 microscope and processed using Adobe Photoshop.

### RNA extraction and RT-qPCR

RNA isolation and quantitative real-time PCR were conducted as previously described (30). Briefly, 2 × 10^5^ conidia/mL of AF1161 or 5 × 10^5^ conidia/mL of ∆*mpkA* were statically incubated in 20 mL RPMI-1640 medium at 37°C for 12 h. The medium was then replaced with fresh RPMI-1640 medium containing either 10 μg/mL PCA or 0.75 mg/mL Rhls, followed by incubation at 37°C for an additional 2 h. Fungal mycelia were harvested by filtration, immediately frozen, and ground in liquid nitrogen. Total RNA was extracted using the Uniq-10 Column Total RNA Purification Kit (B511361, Sangon Biotech) according to the manufacturer’s instructions. Genomic DNA removal and cDNA synthesis were performed using the HiScript II Q RT SuperMix for qPCR (with gDNA Wiper) (R323-01, Vazyme). RT-qPCR was performed using AceQ qPCR SYBR Green Master Mix (Q331-02, Vazyme) on an ABI StepOne Fast Real-Time PCR System (Applied Biosystems). Relative gene expression levels were normalized to the *tub* reference gene and calculated using the 2^⁻ΔΔCT^ method (56).

## Data Availability

The authors confirm that the data supporting the findings of this study are available within the article and its supplementary materials.

## SUPPLEMENTAL MATERIAL

Supplemental material aem.00918-26-s0001.docx Fig. S1 to S6; Tables S1 and S2.

ASM does not own the copyrights to Supplemental Material that may be linked to, or accessed through, an article. The authors have granted ASM a non-exclusive, world-wide license to publish the Supplemental Material files. Please contact the corresponding author directly for reuse.
