# Supplementary material for: Bacterial bioactive metabolites can facilitate cooperative interactions within Aspergillus fumigatus–Pseudomonas aeruginosa mixed biofilms by promoting fungal polysaccharide production
Source: Appl Environ Microbiol. 2026 Jun 15;92(7):e00918-26. doi: 10.1128/aem.00918-26 (PMC13390494; doi:10.1128/aem.00918-26)
Supplement: Supplemental material — Fig. S1 to S6; Tables S1 and S2. [file aem.00918-26-s0001.docx]

**Supplementary Materials**

**Supplementary Figures**

**
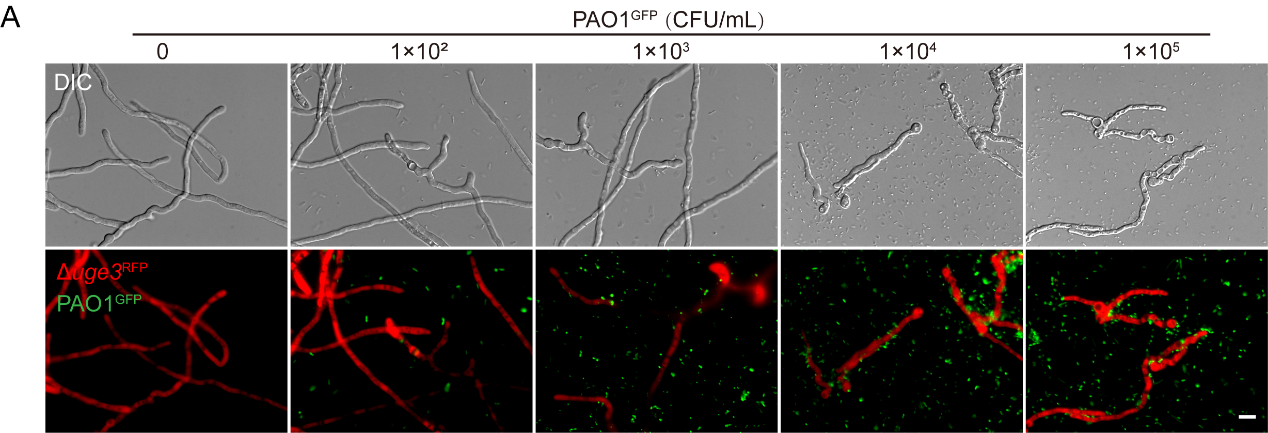
**

**Figure S1 The interaction between *P. aeruginosa* and *A. fumigatus* is dependent on GAG. A.** Representative images showing the growth status of ∆*uge3*^RFP^ co-cultured with PAO1^GFP^ at different concentrations. 1×10^5^ conidia/mL ∆*uge3*^RFP^ and PAO1^GFP^at indicated concentrations were statically cultured in RPMI-1640 medium at 37°C for 12 h, scale bar = 10 μm. All experiments shown above were performed three times.


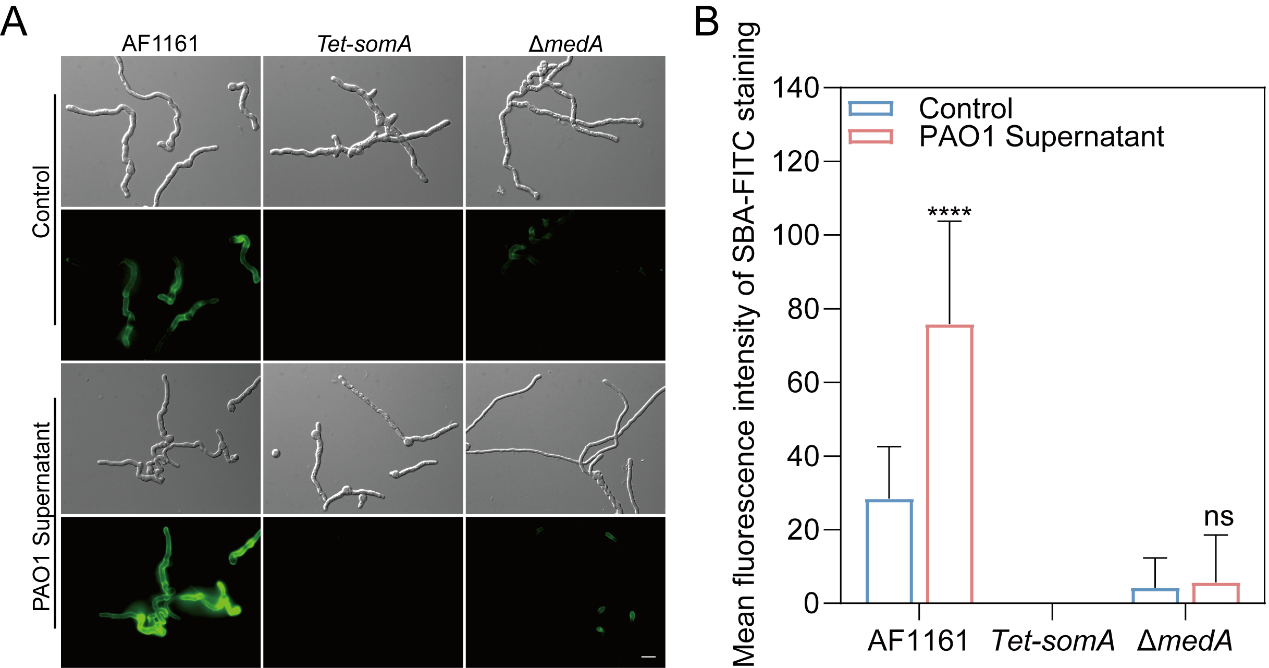


**Figure S2 The production of GAG induced by *P. aeruginosa* depends on GAG biosynthesis regulators. A.** Representative images of SBA-FITC staining of *A. fumigatus* *Tet-somA* and ∆*medA* mutants treated with *P. aeruginosa* supernatant. 1×10^5^ conidia/mL *A. fumigatus* were statically cultured with *P. aeruginosa* supernatant in RPMI-1640 medium at 37°C for 8-12 h, scale bar = 10 μm. **B.** Mean fluorescence intensity of images in panel A. All the above experiments were performed in triplicate. Bars represent mean ± SD. A two-way analysis of variance (ANOVA) along with Šidák multiple comparison tests was utilized for statistical analysis. (****, P<0.0001; ns, no significant difference).


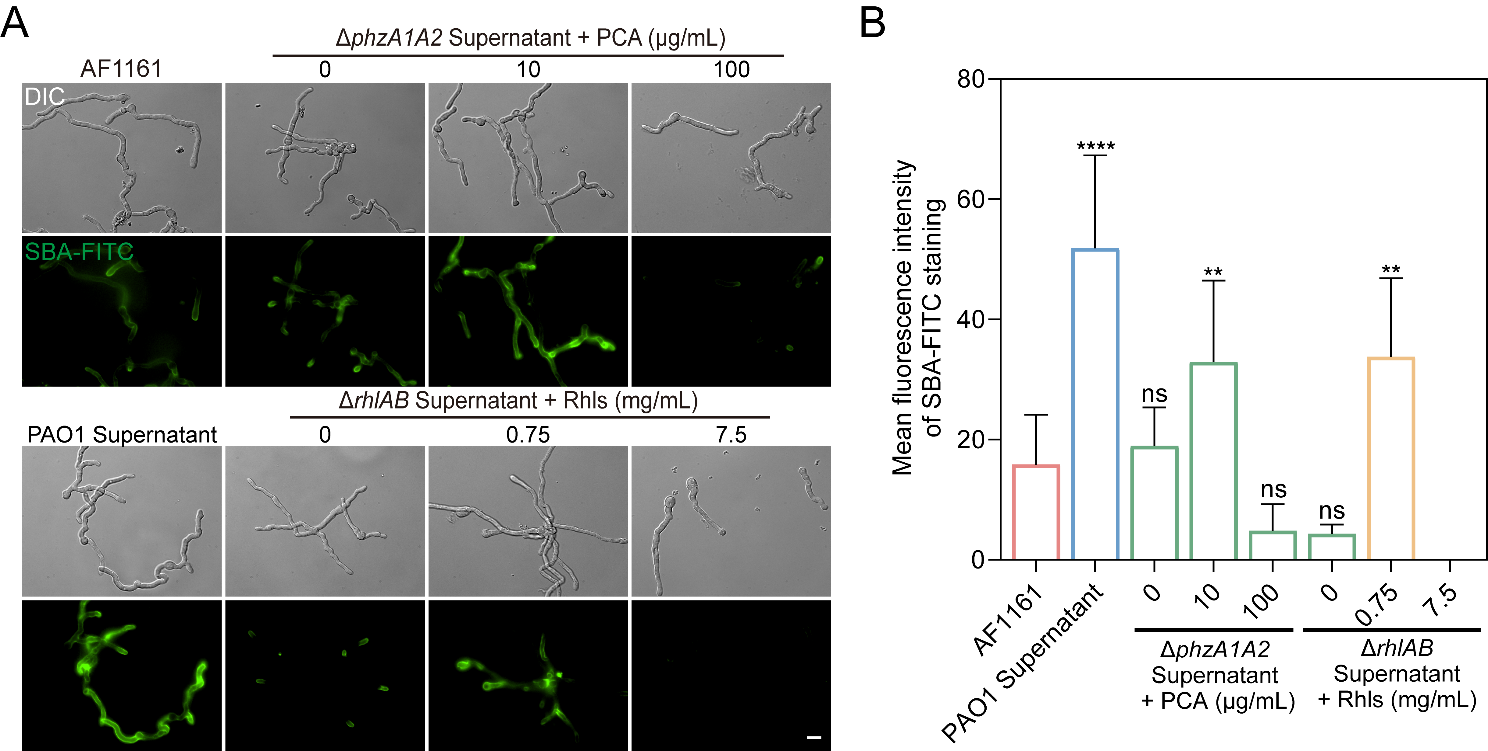


**Figure S3 *P. aeruginosa* secondary metabolites phenazine and rhamnolipids are required for GAG induction in *A. fumigatus.* A.** Representative images of SBA-FITC staining of *A. fumigatus* treated with *P. aeruginosa* ∆*phzA1A2* and ∆*rhlAB* mutants supernatants with PCA and Rhls. 1×10^5^ conidia/mL *A. fumigatus* were statically cultured with *P. aeruginosa* supernatant in RPMI-1640 medium at 37°C for 8-11 h, scale bar = 10 μm. **B.** Mean fluorescence intensity of images in panel A. All the above experiments were performed in triplicate. Bars represent mean ± SD. A one-way analysis of variance (ANOVA) along with Dunnett's multiple comparison tests was utilized for statistical analysis. (**, P<0.01; ****, P<0.0001; ns, no significant difference).


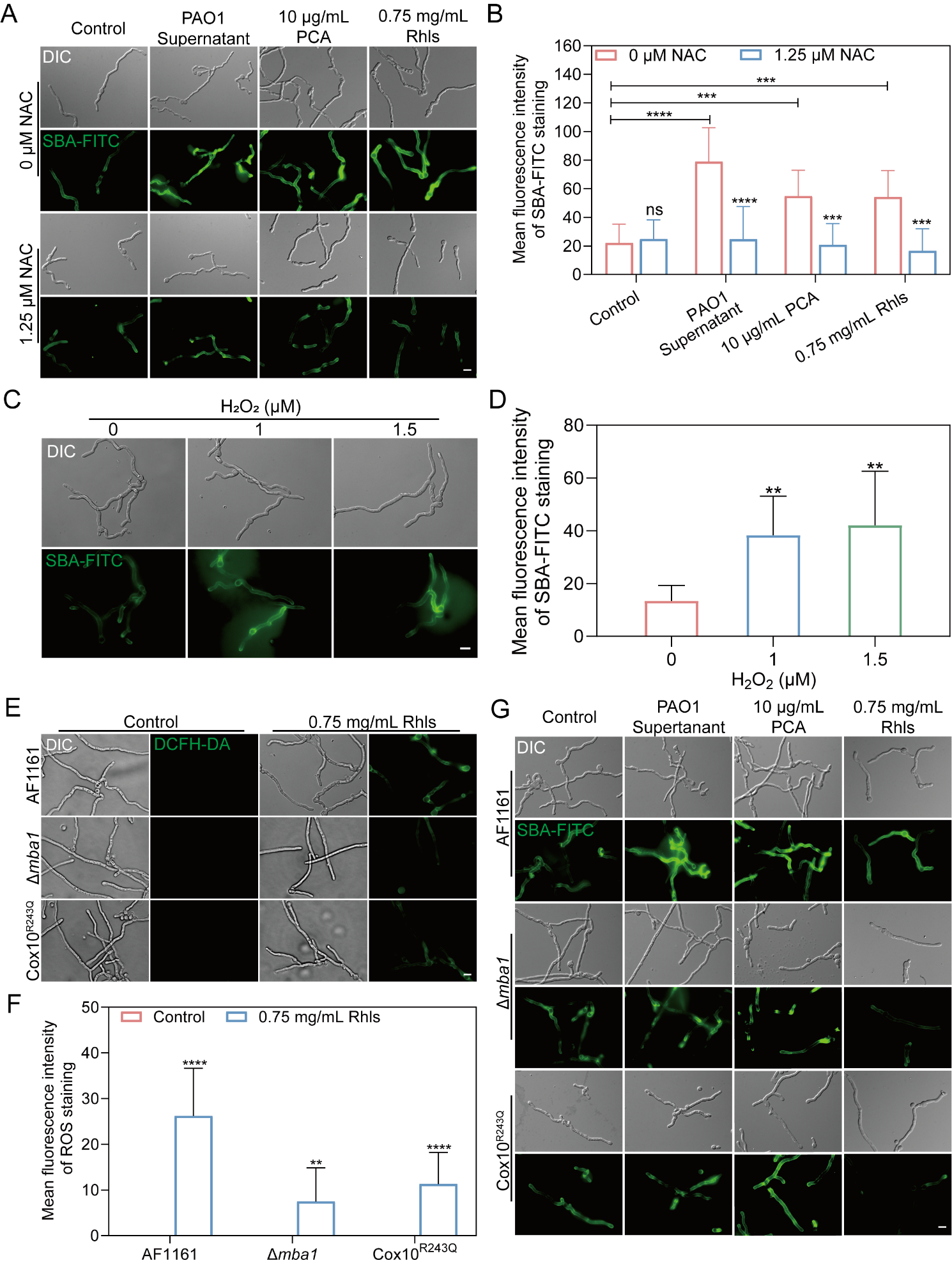


**Figure S4 The promotion of GAG production by *A. fumigatus* via *P. aeruginosa* is dependent on oxidative stress. A.** Representative images of SBA-FITC staining of *A. fumigatus* treated with *P. aeruginosa* supernatant, PCA and Rhls with or without NAC. 1×10^5^ conidia/mL *A. fumigatus* were statically cultured with *P. aeruginosa* supernatant, PCA and Rhls with or without NAC in RPMI-1640 medium at 37°C for 8-10 h, scale bar = 10 μm. **B.** Mean fluorescence intensity of images in panel A. **C.** Representative images of SBA-FITC staining of *A. fumigatus* treated with H_2_O_2_. 1×10^5^ conidia/mL *A. fumigatus* were statically cultured with H_2_O_2_ at indicated concentrations in RPMI-1640 medium at 37°C for 8 h, scale bar = 10 μm. **D.** Mean fluorescence intensity of images in panel C. **E.** Representative images of DCFH-DA staining of *A. fumigatus* ∆*mba1* and Cox10^R243Q^ mutants treated with Rhls. 1×10^5^ conidia/mL *A. fumigatus* ∆*mba1* and Cox10^R243Q^ mutants were statically cultured with Rhls in RPMI-1640 medium at 37°C for 9-14 h, scale bar = 10 μm. **F.** Mean fluorescence intensity of images in panel E. **G.** Representative images of SBA-FITC staining of *A. fumigatus* ∆*mba1* and Cox10^R243Q^ mutants with *P. aeruginosa* supernatant, PCA and Rhls. 1×10^5^ conidia/mL *A. fumigatus* ∆*mba1* and Cox10^R243Q^ mutants were statically cultured with *P. aeruginosa* supernatant, PCA and Rhls in RPMI-1640 medium at 37°C for 8-15 h, scale bar = 10 μm. All the above experiments were performed in triplicate. Bars represent mean ± SD. A one-way analysis of variance (ANOVA) along with Dunnett's multiple comparison tests and two-way ANOVA along with Šidák multiple comparison tests was utilized for statistical analysis. (**, P<0.01; ***, P<0.001; ****, P<0.0001; ns, no significant difference).


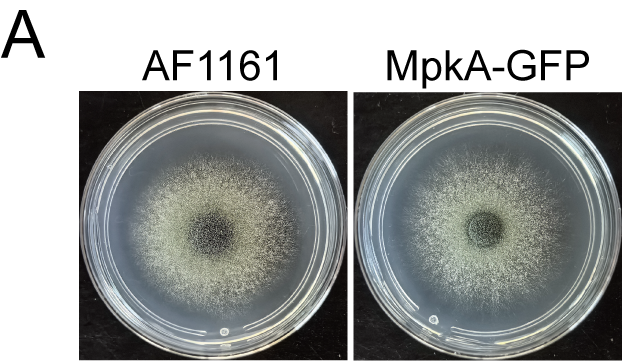


**Figure S5 Phenotypes of the AF1161 and MpkA-GFP strains cultured on MM.** Colony morphology was imaged after 48 h. The above experiments were performed in triplicate.

**
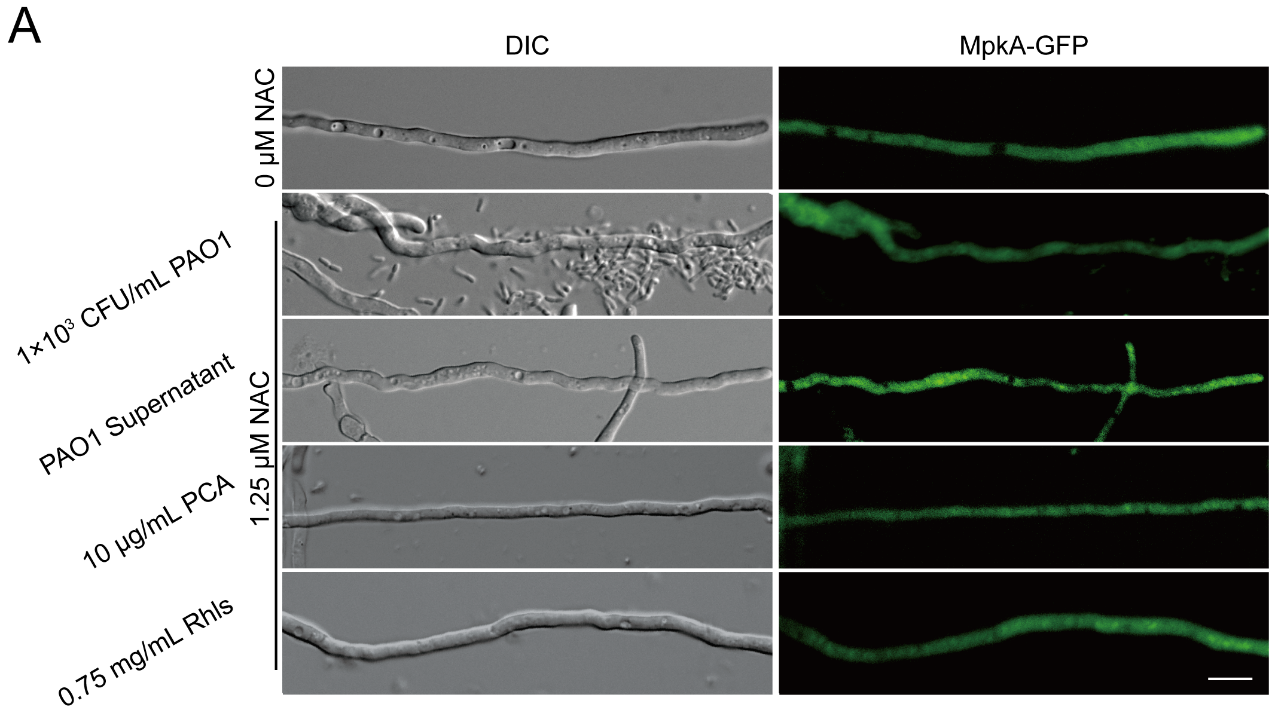
**

**Figure S6 Oxidative stress caused by *P. aeruginosa* is crucial for the MpkA-GFP accumulates in the nucleus.** **A.** Representative images of MpkA-GFP localization under treatment with *P. aeruginosa, P. aeruginosa* supernatant, PCA, Rhls or in combination with NAC*.* 1×10^5^ conidia/mL *A. fumigatus* were incubated with 1×10^3^ CFU/mL *P. aeruginosa* or NAC in RPMI-1640 medium at 37°C for 9-11 h. Scale bar = 10 μm. The above experiments were performed in triplicate.

**Supplementary Tables**

**Supplementary Table 1 Strains used in this study**

| Strain | Genotype | Reference | |
| --- | --- | --- | --- |
| AF1160 | ∆*ku80, pyrG* | | FGSC ^a^ |
| AF1161 | AF1160::*pyrG* | | (1) |
| AF1160^GAPDH-RFP^ | AF1160, *gapdh:: rfp:: pyrG* | | (2) |
| ∆*uge3* | AF1161, ∆*uge3::hph* | | (2) |
| ∆*uge3*^GAPDH-RFP^ | AF1160, *gapdh*::*rfp*::*pyrG*, ∆*uge3*::*hph* | | (2) |
| ∆*mba1* | AF1160, ∆*mba1::pyrG* | | (3) |
| Cox10^R243Q^ | AF1160, ∆*cox10(p)::cox10^R243Q^::pyrG* | | (4) |
| ∆*mpkA* | AF1161, ∆*mpkA::hph* | | This study |
| MpkA-GFP | AF1160, *mpkA::gfp::pyrG* | | This study |
| *Tet-somA* | AF1161*, tet(p)::somA::ptrA* | | (5) |
| ∆*medA* | AF1160*,* ∆*medA::pyrG* | | (5) |
| ∆Af*yap1* | AF1161, ∆Af*yap1::hph* | | (6) |

a, FGSC, Fungal Genetics Stock Center.

1. Jiang H, Shen Y, Liu W, Lu L. 2014. Deletion of the putative stretch-activated ion channel Mid1 is hypervirulent in *Aspergillus fumigatus*. Fungal Genetics and Biology 62:62–70.

2. Cong L, Zhang Y, Chen H, Lu R, Zhang S. 2025. The *Aspergillus fumigatus* extracellular polysaccharide galactosaminogalactan displays context-dependent cooperative and competitive social traits in mixed biofilms. Journal of Fungi 11:695.

3. Zhu G, Chen S, Zhang Y, Lu L. 2023. Mitochondrial membrane-associated protein Mba1 confers antifungal resistance by affecting the production of reactive oxygen species in *Aspergillus fumigatus*. Antimicrob Agents Chemother 67:e00225-23.

4. Li Y, Zhang Y, Zhang C, Wang H, Wei X, Chen P, Lu L. 2020. Mitochondrial dysfunctions trigger the calcium signaling-dependent fungal multidrug resistance. Proc Natl Acad Sci USA 117:1711–1721.

5. Chen Y, Le Mauff F, Wang Y, Lu R, Sheppard DC, Lu L, Zhang S. 2020. The transcription factor SomA synchronously regulates biofilm formation and cell wall homeostasis in *Aspergillus fumigatus.* mBio 11:e02329-20.

6. Furukawa T, van Rhijn N, Fraczek M, Gsaller F, Davies E, Carr P, Gago S, Fortune-Grant R, Rahman S, Gilsenan JM, Houlder E, Kowalski CH, Raj S, Paul S, Cook P, Parker JE, Kelly S, Cramer RA, Latgé J-P, Moye-Rowley S, Bignell E, Bowyer P, Bromley MJ. 2020. The negative cofactor 2 complex is a key regulator of drug resistance in *Aspergillus* *fumigatus*. Nat Commun 11:427.

**Supplementary Table 2 Primers used in this study**

| Name/Purpose | Sequence (5’ to 3’) |
| --- | --- |
| Genes deletion and confirmation | |
| ∆*mpkA*-P1 | GATGGCTTCGCGTTCAGTCTG |
| ∆*mpkA*-P2 | CGACAGGTCTACATTCATGAC |
| ∆*mpkA*-P3 | GGCACCGGTCAACCATGATCTGATCAAAACCGACAGCTTTGTC |
| ∆*mpkA*-P4 | ATGGTTGCCTAGTGAATGCTCCGACGTTGACTTTCGTATGAAGC |
| ∆*mpkA*-P5 | CACATACACAAGCCCACTCCA |
| ∆*mpkA*-P6 | ACCTACCAGCAGATCCTCACT |
| ∆*mpkA*-SF | GTTCATACTGGCGAGGGAG |
| ∆*mpkA*-SR | CAGATGTGCAGGTACGGGTG |
| *hph*-F | AGATCATGGTTGACCGGTGC |
| *hph*-R | CGGAGCATTCACTAGGCAACCAT |
| *hph*-down | GTCACTGTACAGAGCTCACG |
| *hph*-up | GTTGGTGTCGATGTCAGCTC |
| Reporter strains construction and confirmation | |
| MPKA-P1 | TAGGATCATCGATGGAGTGTCC |
| MPKA-P2 | TCGTCTTCACTCTCGTTGGC |
| MPKA-P3 | CCAGCGCCTGCACCAGCTCCTTGGACATCCATCCCCCG |
| MPKA-P4 | CATCAGTGCCTCCTCTCAGACAGACGTTGACTTTCGTATGAAGCTTG |
| MPKA-P5 | TACATGGTTGGTTACGATTGGATG |
| MPKA-P6 | ACTCATGGCATTCAATGGAATG |
| GFP+*pyrG*-F | GGAGCTGGTGCAGGCGCTGG |
| GFP+*pyrG*-R | CTGTCTGAGAGGAGGCACTGATG |
| GFP+*pyrG*-down | GTGAAGAGCATTGTTTGAGGC |
| GFP+*pyrG*-up | GATACAGGTCTCGGTCCCTA |
| Quantitative PCR | |
| qPCR-*tubA*-F | TCCCAACAACATCCAGACCG |
| qPCR-*tubA*-R | TGATCACCGACACGCTTGAA |
| qPCR-*somA*-F | TAGCCCTTCATCACTCACGC |
| qPCR-*somA*-R | GCCATAGTAGTCGCCGTTCA |
| qPCR-*medA*-F | TATTATGGGCTTTCCCGCCC |
| qPCR-*medA*-R | AGTGTCATACCCCGATTGGC |
| qPCR-*stuA*-F | CTACTTTGAGCAACACGCGG |
| qPCR-*stuA*-R | AGGTGGAACTGATTGGCGTT |
| qPCR-*uge3*-F | GCTGTTAGCCTCCCAGTACC |
| qPCR-*uge3*-R | GGACTTGGTCGTACCCCAT |
| qPCR-*agd3*-F | ACGCGGACGTCTTCAAGGAG |
| qPCR-*agd3-*R | GTTGTGCAGACCGGTGATGG |
| qPCR-*ega3*-F | ACGACAAGTCCACCATCGCA |
| qPCR-*ega3*-R | GCCCAGATCCGAGTCCTTGA |
